# Precision formulation, a new concept to improve dietary amino acid absorption based on the study of cationic amino acid transporters

**DOI:** 10.1016/j.isci.2024.108894

**Published:** 2024-01-14

**Authors:** Guillaume Morin, Karine Pinel, Cécile Heraud, Soizig Le-Garrec, Chloé Wayman, Karine Dias, Frédéric Terrier, Anthony Lanuque, Stéphanie Fontagné-Dicharry, Iban Seiliez, Florian Beaumatin

**Affiliations:** 1 Université de Pau et des Pays de l’Adour, E2S UPPA, INRAE, NUMEA, Saint-Pée-sur-Nivelle, France

**Keywords:** Biological sciences, Physiology, Animal nutrition, Aquaculture nutrition

## Abstract

Amino acid (AA) transporters (AAT) control AA cellular fluxes across membranes, contributing to maintain cellular homeostasis. In this study, we took advantage of rainbow trout metabolic feature, which highly relies on dietary AA, to explore the cellular and physiological consequences of unbalanced diets on AAT dysregulations with a particular focus on cationic AAs (CAA), frequently underrepresented in plant-based diets. Results evidenced that 24 different CAAT are expressed in various trout tissues, part of which being subjected to AA- and CAA-dependent regulations, with *y+LAT2* exchanger being prone to the strongest dysregulations. Moreover, CAA were shown to control two major AA-dependent activation pathways (namely mTOR and GCN2) but at different strength according to the CAA considered. A new feed formulation strategy has been put forward to improve specifically the CAA supplemented absorption in fish together with their growth performance. Such “precision formulation” strategy reveals high potential for nutrition practices, especially in aquaculture.

## Highlights

• Dietary glycine supplementation decreases fish feed intake but preserves fish growth
• Cationic amino acids (CAA) availabilities tightly control mTOR and GCN2 activities
• GCN2 pathway regulates the expression of CAA transporters, including y^+^LAT2 exchanger
• Glycine supplementation improves CAA bioavailability likely through exchange mechanism

## Graphical abstract

**Figure:**
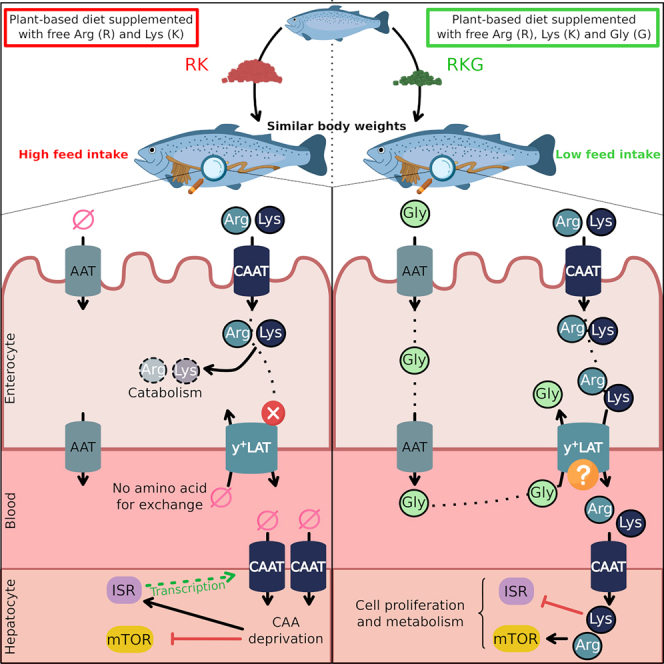

## Introduction

In most organisms, if not all, amino acids (AA) constitute basic, but crucial, molecules for life. The past decades have demonstrated that AA functions are not only supported by their roles as building blocks for protein synthesis but also by their abilities fueling numerous metabolic pathways as well as their capacities to control homeostasis as signaling molecules. Indeed, while most of the 20 natural proteinogenic AA serves as precursors for energy, nucleotide, lipid and glucose metabolisms,^1^ a significant proportion also bears signaling functions,^2^ directly^3^^,^^4^ or indirectly through their related secondary metabolites.^5^^,^^6^ Therefore, the term “functional” AA has been recently introduced^7^ to complete the conventional AA classification, established on nutrient requirements determined only with respect for growth performance and/or nitrogen balance of species, into essential (EAA), non-essential (NEAA) and conditionally essential AA (CEAA). Thus, “functional AA” are defined as “those AA that participate in and regulate key metabolic pathways to improve health, survival, growth, development, lactation, and reproduction of the organisms.” Accordingly, this new concept reshuffles the deck and sheds new light on the countless functions supported by AA.

Conscious of the massive input of AA on cellular homeostasis and organism physiology, much efforts have been made by the scientific community to understand the molecular mechanisms controlling AA functions in cells. Among these, the absorption of AA has received a particular attention since it constitutes the first limiting step for an AA to get functional.^8^ In past decades, amino acid transporters (AAT) were described and classified into 16 different systems^9^ according to the nature of the AA transported (Neutral; Cationic or Anionic) together with their dependencies onto environmental conditions (e.g., pH or ions). Recently, with technical advances made in molecular and genomic analysis, the landscape of AAT appeared to be even more complex since a greater number of AAT were identified in genome species.^2^ For instance, more than 60 AAT genes, nowadays grouped in the super gene family of SoLute Carriers (SLC) based on sequence homologies, were identified in humans (www.bioparadigms.org). When compared to the number of natural AA to uptake, this impressive number of different AAT witnesses the challenges required to maintain AA homeostasis in an organism especially when considering that all organs or cell types do not display the same metabolic needs. Indeed, beside the need to fine-tune, qualitatively and quantitatively, the AA fluxes across biological membranes, the large number of AAT also allows tissue specific expression as well as different intracellular location (such as mitochondria, plasma membrane or lysosomes) to finally support specific or ubiquitous cellular functions mentioned above. Accordingly, it is therefore not surprising that even a unique dysregulation of a said AAT expression can induce cellular disorders. Whether these dysregulations lead to a defect, or conversely, to a stimulation of AA absorption/excretion, most of them are associated with pathologies and diseases such as aminoaciduria,^10^ obesity,^11^ hypertension^12^ and cancer.^13^ This emphasizes even more the complexities of the underlying mechanisms required to tightly control AAT expressions and functions in an organism to finally preserve homeostasis and keep cellular disorders at bay.

Among the factors leading to the above-mentioned pathologies, nutrition has received particular attention in recent years. Indeed, several studies have highlighted the need to reduce red meat consumption in high-income Western countries in order, for instance, to prevent the onset of cancers and cardiovascular diseases.^14^ As a consequence, an increase consumption of plant-based diets (PBDs) to replace, fully or not, the consumption of any animal-derived products is observed. If such practice has already shown to bring multiple benefits for human health,^15^ it requires a careful attention notably to overcome the so called “low quality protein” of PBDs together with low plant protein digestibility. Indeed, it is known that, compared to animal proteins, plant proteins display an unbalanced AA composition for which methionine, lysine, arginine and threonine are frequently underrepresented that could lead to dietary restrictions for those AA. Moreover, such issue can be amplified by the decrease of protein digestion caused by antinutritional factors brought in PBDs.^16^ Therefore, if such negative criteria are no longer a matter of debate for human health, their impact in metabolic dysregulations remains to clarify.^17^ Interestingly, the replacement of animal proteins by plant proteins is, for ecological and economic reasons, also one of the main goals of the aquaculture field.^18^ Indeed, the replacement of fishmeal (FM) by other protein sources in aquafeeds, such as plant proteins, appears as the most convenient alternative to preserve natural resources currently threatened by overfishing activities. Nonetheless, such replacement is not without consequences in fish biology since disorders are observed, especially in carnivorous fish such as in RT when fed 100% PBD, among which growth retardation. Since the metabolism of carnivorous fish mainly relies on dietary protein (45% of dietary protein required compared to 15% in human nutrition), and so on AA, part of the disorders observed are suspected to be caused by the “low quality” of plant proteins. In this regard, an in-depth analysis of transcriptomic data from RT fed FM-based diets or PBD published in two independent studies,^19^^,^^20^ led us to note that *ddit3* (*DNA Damage Inducible Transcript 3*, also known as *chop*), was dysregulated in the latter group. Although several pathways were shown to control *DDIT3* expression, the General Control Nonderepressible 2 (GCN2) pathway appeared as a likely hypothesis that could explain *ddit3* dysregulation in fish fed PBD. Indeed, upon AA restriction/starvation, uncharged tRNAs interact with the GCN2 serine/threonine kinase leading to its activation. Consequently, GCN2 phosphorylates eukaryotic initiation factor 2 (eIF2) α which in turn represses the general protein translation and promotes the expression of several transcription factors, including *ddit3* and several activating transcription factors (ATF).^21^^,^^22^^,^^23^ Consequently, transcription factors induce the expression of several metabolic genes as well as AA transporters, including some CAAT transporters as described in mammals,^2^^,^^24^ to help cells to better cope with AA starvation conditions. Interestingly, we also noticed from two studies above mentioned that two cationic AAT (CAAT) namely *cat2* (encoded by SLC7A2 gene) and *y*^*+*^*lat2* (a heterodimeric CAA exchanger encoded by 4F2hc (SLC3A2) and SLC7A6 genes) were also dysregulated in fish fed PBD. Thus, we wondered if these dysregulations could reflect that PBD do not cover well the metabolic requirements of fish with respect for CAA, despite being supplemented in their purified forms (according to the nutrient requirements established in fish^25^), leading to negative outcomes on fish growth performance. This hypothesis was indeed supported by the fact that (1) the AA restriction-induced GCN2 activation is conserved and active in rainbow trout cells^26^ and (2) the activation of this pathway is described in mammals to derepress CAAT expression to favor AA absorption upon AA limiting conditions.^27^

Therefore, investigations were performed in order to determine whether specific CAA restriction could affect transcriptional regulations of CAAT as well as two key AA-sensing pathways such as GCN2 and the mechanistic Target Of Rapamycin (mTOR), known to control cellular homeostasis and proliferation. For such purpose, two important points had to be considered: First, it was essential to identify and characterize the whole sub-family of CAAT genes in trout, since, to the best of our knowledge, only a few CAAT were previously studied in RT.^28^^,^^29^ Then, since hormones and cytokines are known regulators of AAT expression as well, it seems particularly crucial to avoid systemic effects to be able to reveal the specific AA-dependent AAT regulations. So, the use of RT cell lines appeared much convenient, besides important ethical considerations, since the use of *in vitro* approaches allows to perfectly control cellular environment, in particular by specifically depleting on-demand growing media for AA or CAA. Therefore, a full set of experimentations (including *in silico*, RTqPCR and western blot analysis as well as cell proliferation assays) has been conducted in two different cell lines available from the RT invitrome.^30^ Results obtained clearly identified the family of CAAT expressed in trout tissues as well as demonstrated that part of these CAAT are subjected to AA and CAA dependent regulations, certainly through GCN2-dependent mechanisms. Moreover, results obtained disclosed differences in GCN2 and mTOR pathway activations together with strong outcomes on cellular proliferation capacities with respect for the nature of the CAA considered. Moreover, *in vitro* results gathered allowed us to test and confirm, via an *in vivo* experiment, a new nutritional strategy. Such strategy relies on a minor but precise adjustment of the dietary AA composition that aims to greatly improve the specific absorption of CAA supplemented to PBD, leading to positive effects on fish growth performance. Finally, the use of the *in vitro* approach, through the characterization of two independent RT cell lines, revealed features that, beyond insightful knowledge gathered for agronomy, could also serve medical science.

## Results

### In silico identification of the whole sub-family of CAAT genes and validation of their expressions in RT tissues and cell lines

**Figure 1:**
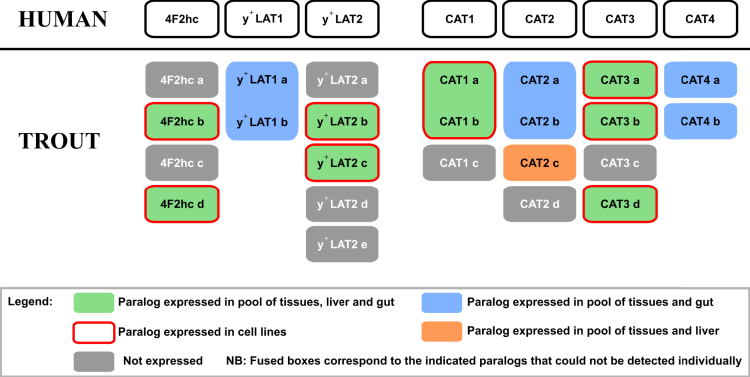
Human genes coding for y^+^LAT and CAT transporters and their ortholog genes identified in RT genome and expressed in RT tissues and in two RT cell lines (RTH-149 and RTgutGC) 24 orthologs of these 7 human CAAT-related genes were identified following *in silico* analysis of RT genome (Omyk 1.0). When possible, RT-qPCR primers were designed to discriminate each paralog. Primers that recognize more than one paralog are shown as fused boxes for the indicated paralogs. Gene expression was assessed in a pool of RT tissue samples (including liver, gut, muscle, kidney, ovary and brain) and in liver or gut samples separately as well as in the two RT cell lines. Gene expression analysis by RT-qPCR in RTH-149 and RTgutGC cell lines revealed that they express the same CAATs also found to be expressed in liver and gut tissues.

When compared to mammals, RT undergone two supplementary whole-genome duplication (WGD) events, namely the teleost-specific- and salmonid-specific WGD, which considerably increased the number of paralogs for a given mammalian gene.^31^ Thus, we performed an *in silico analysis* to identify all the CAAT genes present in RT genome on the basis of protein sequence homologies to human CAAT (Table 1). Interestingly, results presented in Figure 1 showed that for each of the 7 human CAAT genes coding for y^+^LAT and CAT transporters at least two paralogs have been found in RT genome, with *y*^*+*^*LAT2* displaying up to 5 paralogs. Moreover, and with the unique exception of *ORNT2* gene, all the other CAAT known in mammals were also found in RT genome (Figure S1). Since it was previously shown that all duplicated genes were not expressed or functional in RT,^31^ expression levels of the 42 different CAAT paralogs identified were assessed through RT-qPCR analysis performed in the RT tissues studied including gut, liver, kidney, muscle, brain and ovary. Consistently with previous findings according which around 50% of duplicated genes are expressed,^31^ at least 24 RT CAAT genes are expressed in RT tissues (Figures 1 and S1) covering the expression of each human CAAT ortholog with at least one RT paralog. Meanwhile, in the two RT cell lines monitored, namely RTH-149 and RTgutGC cells, 16 CAAT paralogs were detected. It is interesting to observe that these two cell lines expressed the same and exact set of CAAT paralogs while they originate from different fish individuals and tissues as well as the fact that they were established following either “natural” immortalization or independent *ex vivo* immortalization protocols performed in distinct laboratories. Altogether, this particular feature offered an opportunity to study the nutritional regulations of these 16 RT CAAT genes in two independent cell lines, allowing therefore to decipher universal nutrient-dependent CAAT regulatory mechanisms from potential cell line-dependent mechanisms.

**Table 1:** Cationic amino acid transporter paralog genes identified in rainbow trout genome

Human transporter	Human gene	Number of paralog genes found in RT genome	Chromosome	Gene ID	Attributed name	Expression detected in RT tissues or cell lines
CAT1	SLC7A1	3	10	110533230	*cat1 a*	Yes
			10	110533232	*cat1 b*	Yes
			27	110507241	*cat1 c*	No
CAT2	SLC7A2	4	14	110489197	*cat2 a*	Yes
			25	110504480	*cat2 b*	Yes
			25	110504711	*cat2 c*	Yes
			29	110510086	*cat2 d*	No
CAT3	SLC7A3	4	10	110534152	*cat3 a*	Yes
			14	110488786	*cat3b*	Yes
			25	110504989	*cat3 c*	No
			29	110510151	*cat3 d*	Yes
4F2hc	SLC3A2	4	10	110533969	*4f2hc a*	No
			21	110500797	*4f2hc b*	Yes
			27	110507427	*4f2hc c*	No
			29	110507427	*4f2hc d*	Yes
y^+^LAT2	SLC7A6	5	6	110526237	*y*^*+*^*lat2 a*	No
			8	110530061	*y*^*+*^*lat2 b*	Yes
			14	110487934	*y*^*+*^*lat2 c*	Yes
			18	110496550	*y*^*+*^*lat2 d*	No
			26	110530061	*y*^*+*^*lat2 e*	No
y^+^LAT1	SLC7A7	2	9	110500506	*y*^*+*^*lat1 a*	Yes
			21	110531642	*y*^*+*^*lat1 b*	Yes
rBAT	SLC3A1	1	20	110499302	*rbat*	Yes
b^(0,+)^AT1	SLC7A9	3	19	110497769	*b*^*(0,+)*^*at1 a*	No
			25	110505695	*b*^*(0,+)*^*at1 b*	No
			Unplaced scaffold	110510393	*b*^*(0,+)*^*at1 c*	Yes
ATB^0+^	SLC6A14	1	7	110528352	*atb*^*0,+*^	Yes
SNAT4	SLC38A4	2	15	110490512	*snat4 a*	Yes
			21	110499957	*snat4 b*	Yes
SLC38A9	SLC38A9	1	5	110523173	*slc38a9*	Yes
ORNT1	SLC25A15	3	10	110534225	*ornt1 a*	No
			27	110507247	*ornt1 b*	No
			27	110508105	*ornt1 c*	Yes
ORNT3	SLC25A29	2	4	110522494	*ornt3 a*	No
			19	110498176	*ornt3 b*	Yes
PQLC2	SLC66A1	2	9	110532347	*pqlc2 a*	Yes
			16	110492259	*pqlc2 b*	Yes
CAT4	SLC7A4	2	6	110525572	*cat4 a*	Yes
			11	110535941	*cat4 b*	Yes
SLC7A14	SLC7A14	3	7	110527386	*slc7a14 a*	Yes
			7	110527387	*slc7a14 b*	No
			15	110490917	*slc7a14 c*	Yes

RT paralogs are named according to the number of chromosome where the genes are located in ascending order. In case of different paralogs expressed on the same chromosome, gene names were attributed according to gene ID number in ascending order. Their expressions were assessed by RT-qPCR in a pool of tissues including intestine, liver, muscle, ovary and kidney or a pool of brain for SLC7A4 and SLC7A14 paralogs. Finally, their expression was assessed separately in whole intestine, liver and in the two RT cell lines.

### Total amino acid starvation induced the de-repression of CAAT paralogs in RT cell lines

**Figure 2:**
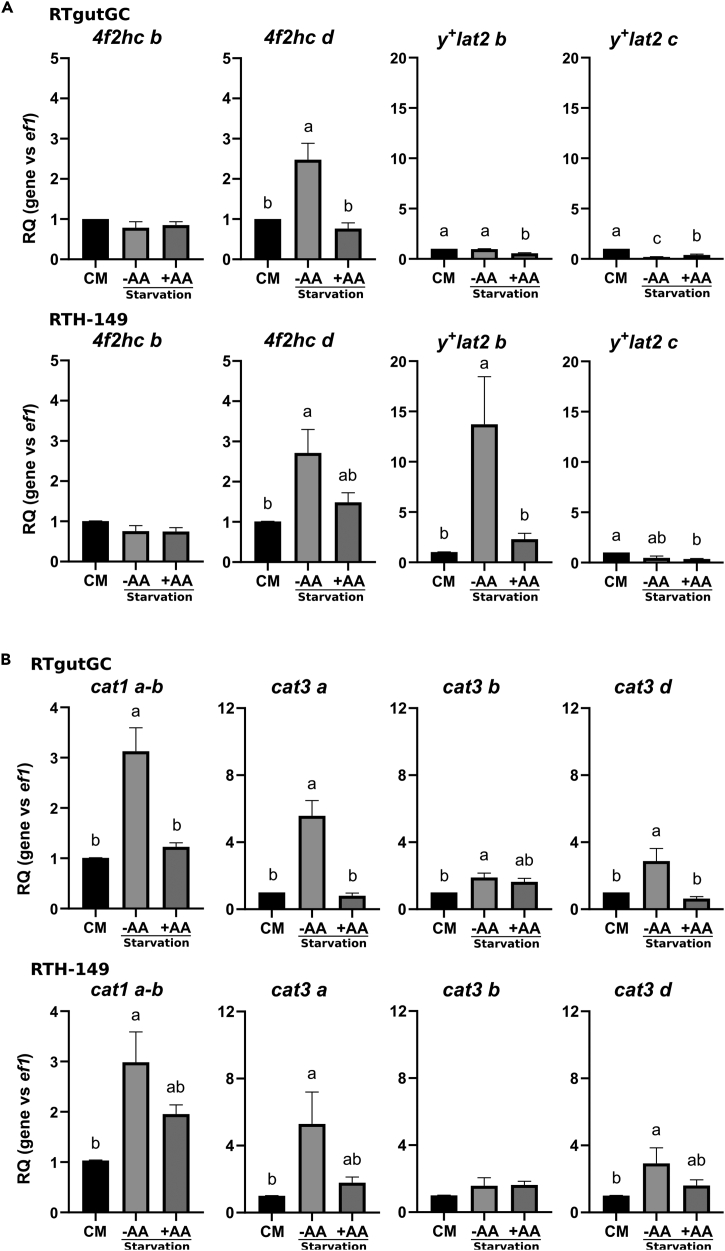
Amino acid dependent regulations of y^+^LAT and CAT transporter genes Amino acid dependent regulations of y^+^LAT (A) and CAT (B) transporter genes in RTgutGC and RTH-149 cells. Cells were grown in complete medium (CM) or in HBSS (starvation) supplemented (+AA) or not (−AA) with amino acids prior to proceed to RNA extraction and RT-qPCR analysis of mRNA levels of CAT and y^+^LAT transporter genes. Results are represented as relative quotient (RQ) normalized on *ef1α* mRNA levels compared to CM. Data are presented as mean ± SEM, *N* = 6 (RTH-149) or *N* = 4 (RTgutGC). Conditions showing results statistically different from each other are indicated using a different letter (p < 0.05, one-way ANOVA Tukey’s post-hoc test).

As previously mentioned, plant-derived proteins do not fulfill the amino acid requirements defined for RT.^25^ PBD are thus supplemented with crystalline amino acids such as K and R to cover the metabolic needs of RT, as performed in the feeding trial later described in this study (See Tables 2 and S2). However, we hypothesized that PBD do not cover well the metabolic AA needs of RT despite being supplemented with AA to re-balance the lack of certain AA in plant protein amino acid compositions. If correct, this hypothesis would imply that the AA-sensing GCN2 pathway should get activated which in turn would lead to the upregulation of GCN2-target genes involved notably in AA synthesis and transport. Based on our previous experience and tools developed to study the AA-induced GCN2 activation in RTH-149 cell line,^26^ we first challenged our hypothesis in RTH-149 and RTgutGC cells with respect for the outcomes of total AA starvation on the expression levels of CAAT paralogs. Results shown in Figures 2 and Figure S2 confirmed that, as previously described in other organisms, part of RT *y*^*+*^*lat* (Figure 2A) and *cat* (Figure 2B) paralogs can be specifically regulated by AA such as *4f2hc d*, *cat1 a-b*, *cat3a*, *cat3d* and *y*^*+*^*lat2 b*. Finally, when the other RT CAAT paralogs were assessed (Figure S2), only *ornt1 c*, a mitochondrial CAAT, and *pqlc2 a*, a lysosomal CAAT, showed a specific AA-dependent response trend, consistent in the two cell lines tested. The other paralogs displayed responses to AA availability that appeared to be more cell line specific. Altogether, these results corroborated previous observations made in other species and confirmed that, in RT cells, some specific CAAT paralogs are up-regulated following AA starvation.

**Table 2:** Diet compositions

Ingredients (% of diet)	RK	RKG
Corn gluten	55	55
Wheat gluten	5	5
Whole wheat	10	10
Rapeseed oil	10.8	10.8
Linseed oil	4	4
Palm oil	2	2
DHA oil (*Schizochytrium* sp.)^a^	0.5	0.5
Rapeseed lecithin	2	2
Mineral premix^b^	4	4
Vitamin premix^c^	1	1
L-Arginine	0.5	0.5
L-Lysine	2.2	2.2
L-Glycine	–	3
Cellulose	3	–
Dry matter (DM, % diet)	93.3	94.9
Crude protein (% DM)	47.6	49.5
Crude fat (% DM)	19.0	22.3
Gross energy (kJ g-1 DM)	24.6	24.9

a Omegavie DHA 700 algae sensor oil (min 70%): Oil from the micro-algae Schizochytrium sp., natural mixed tocopherols E306, vegetable oil (MCT), sunflower lecithin E322, ascorbyl palmitate E304, rosemary extract E392. This oil contains marine DHA Omega 3 fatty acids under Triglycerides form. From POLARIS, Quimper, France.

b Mineral premix: (g or mg kg− 1 diet): dibasic calcium phosphate (20% Ca, 18% P), 35 g; calcium carbonate (40% Ca), 2.15 g; magnesium oxide (60% Mg), 1.24 g; potassium chloride (52% K), 0.9 g; sodium chloride, 0.4 g; zinc sulfate (36% Zn), 0.4 g; ferrous sulfate (20% Fe), 0.2 g; copper sulfate (25% Cu), 0.3 g; manganese sulfate (33% Mn), 0.3 g; sodium selenite (46% Se), 3 mg; cobalt chloride (25% Co), 2 mg; sodium fluoride (45% F), 1 mg; potassium iodide (76% I), 0.4 mg (UPAE, INRAE).

c Vitamin premix: (IU or mg kg−1 diet): retinyl acetate, 5,000 IU; DL-cholecalciferol, 2500 IU; DL-α tocopherol acetate, 50 IU; sodium menadione bisulphate, 10 mg; thiamin, 1 mg; riboflavin, 4 mg; niacin, 10 mg; calcium pantothenate, 20 mg; pyridoxine, 3 mg; biotin, 0.2 mg; folic acid, 1 mg; cyanocobalamin, 0.01 mg; L-ascorbyl-2-polyphosphate, 50 mg; myo-inositol, 300 mg; choline chloride, 1000 mg. All ingredients were diluted with α-cellulose. (UPAE, INRAE).

### GCN2 activation leads to the upregulation of RT CAAT paralogs

**Figure 3:**
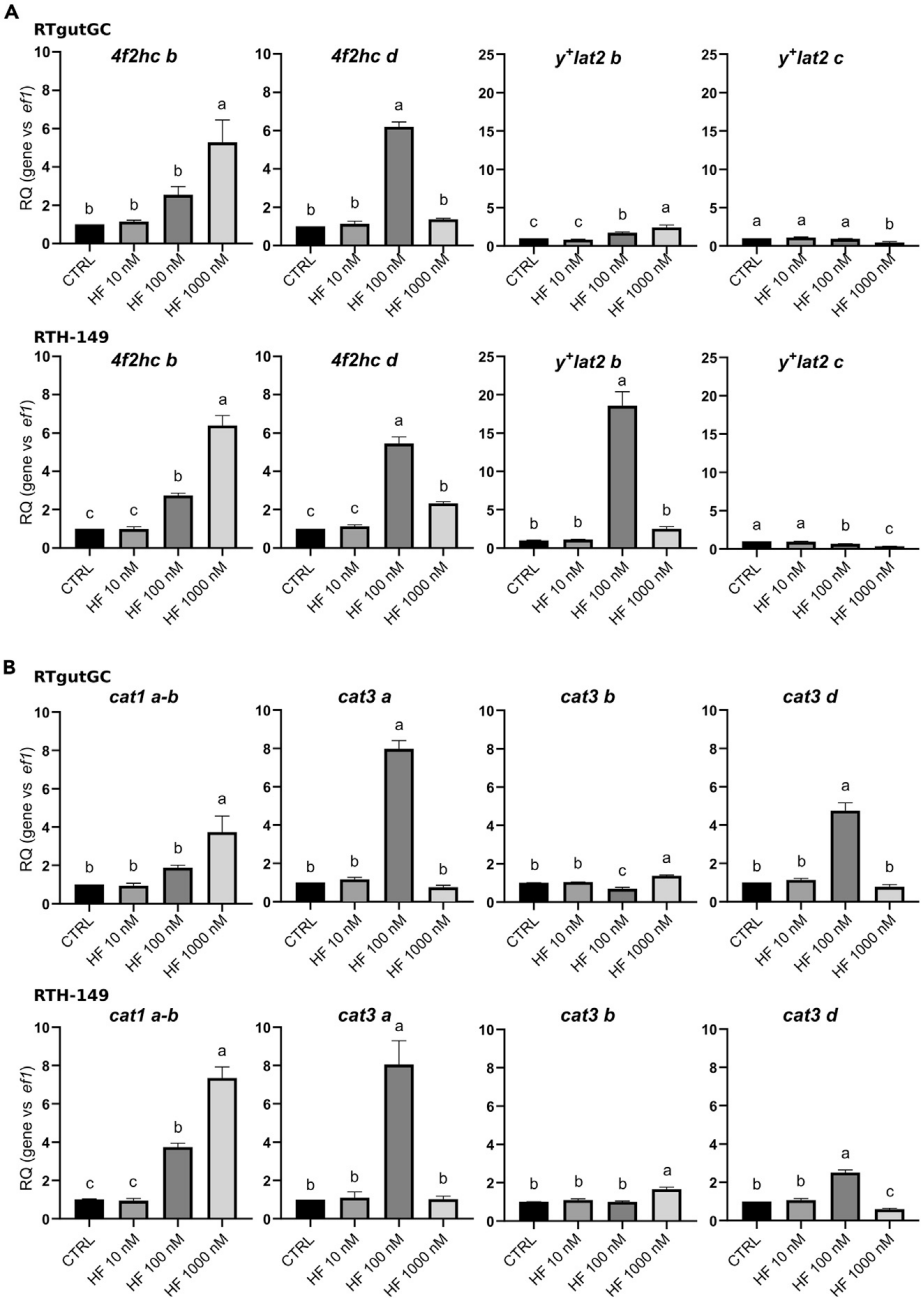
Halofuginone dependent regulations of y^+^LAT and CAT transporters Expression of y^+^LAT (A) and CAT (B) transporter genes induced by halofuginone hydrobromide (HF) in RTgutGC and RTH-149 cells. Cells were grown in complete medium supplemented with indicated concentrations of HF or without (CTRL) prior to proceed to RNA extraction and RT-qPCR analysis of mRNA levels of CAT and y^+^LAT transporter genes. Results are represented as relative quotient (RQ) normalized on *ef1α* mRNA levels compared to CTRL. Data are presented as mean ± SEM, *N* = 4. Conditions showing results statistically different from each other are indicated using a different letter (p < 0.05, one-way ANOVA Tukey’s post-hoc test).

Based on our working hypothesis together with the first results obtained, the involvement of the GCN2 pathway in the up-regulation of RT CAAT paralogs was assessed in the two cell lines tested. To do so, cell lines were grown in presence of halofuginone hydrobromide (HF), a GCN2 activator already described to be effective in RTH-149 cells,^26^ in order to confirm that HF treatments were efficient to induce the expression of ISR target genes from the GCN2 (*ddit3*, *asns*, and *xbp1)* and the PERK-dependent unfolded protein response (UPR) pathways *(edem1 and grp78 genes)* (Figure S3A). We found that below 1μM of HF treatment, none of the two specific UPR target genes (*edem1* and *grp78*) were up-regulated, suggesting that for up to 100 nM of HF, the upregulations observed for *ddit3*, *asns*, and *xbp1* mainly rely on the activation of the GCN2 pathway but not on the PERK axis. Meanwhile, as shown in Figures 3 and S3B, we observed that all the RT CAAT paralogs previously identified to be specifically regulated by AA availability were also significantly up-regulated upon HF treatments in RT cell lines. However, two different expression profiles, exemplified by *4f2hc b* and d paralogs, were observed, suggesting different regulatory mechanisms according to the strength of GCN2 activation. Indeed, it was described that strong GCN2 activation led to a massive upregulation of *ddit3* gene therefore involved in a negative feedback loop in charge to dampen cell survival GCN2 response and to promote cell death.^23^^,^^32^ In this regard, *4f2hc d*, *cat3a* and *cat3d* paralogs (and *y*^*+*^*lat2 b* when considering RTH-149 cells response) displayed an upregulation upon moderate GCN2 activation (100 nM HF) while an increase in HF concentration (1000 nM) led to a repression of this activation. On the other hand, all other CAAT observed to be positively regulated by HF followed expression levels gradually increasing with increase in HF concentrations. Finally, since both starvation condition and HF treatments can also promote ER stress leading to ISR activation through the PERK axis, we investigated whether upon the starvation condition tested an activation of the ISR/PERK-dependent axis could be detected, using tunicamycin (Tun) treatment as a positive control for the induction of ER stress (Figure S4). Consequently, we could observe that, upon starvation or 100 nM HF treatment, RT CAAT paralog dysregulations were not relying on PERK activation since *edem1* and *grp78* genes were only up-regulated upon Tun treatment. Altogether, these results strongly suggest, if not demonstrate, that CAAT are prone to regulations in a GCN2-dependent starvation-induced manner.

### Arginine and/or lysine starvation are enough stimuli to activate GCN2 and promote RT CAAT paralog upregulations

**Figure 4:**
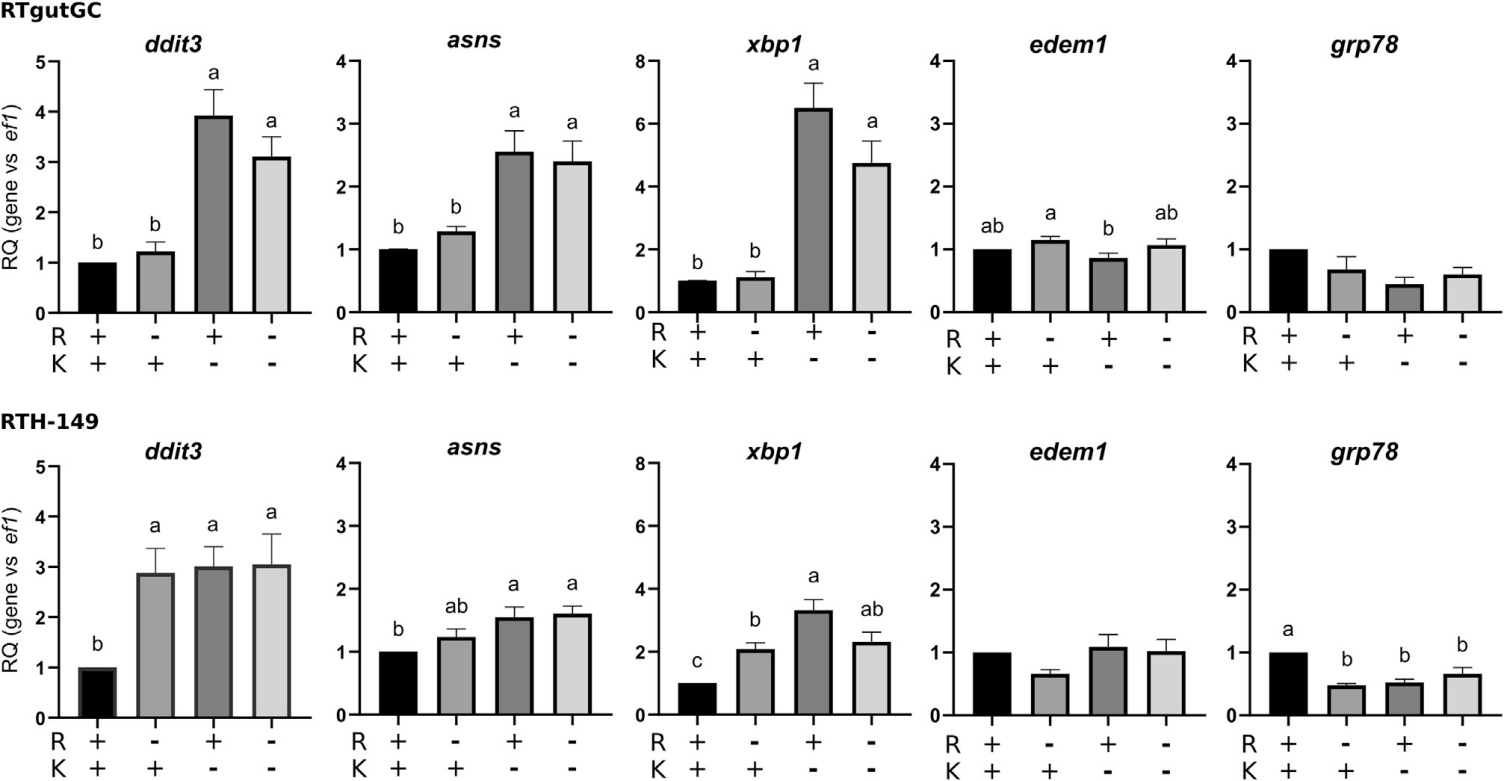
Arginine and lysine dependent regulations of ISR target genes in RTH-149 and RTgutGC cells Cells were grown in arginine (R) and lysine (K) deprived medium supplemented (+) or not (−) with R and K prior to proceed to RNA extraction and RT-qPCR analysis of mRNA levels of CAT and y^+^LAT transporter genes. Results are represented as relative quotient (RQ) normalized on *ef1α* mRNA levels compared to condition with R and K. Data are presented as mean ± SEM, *N* = 6. Conditions showing results statistically different from each other are indicated using a different letter (p < 0.05, one-way ANOVA Tukey’s post-hoc test).

**Figure 5:**
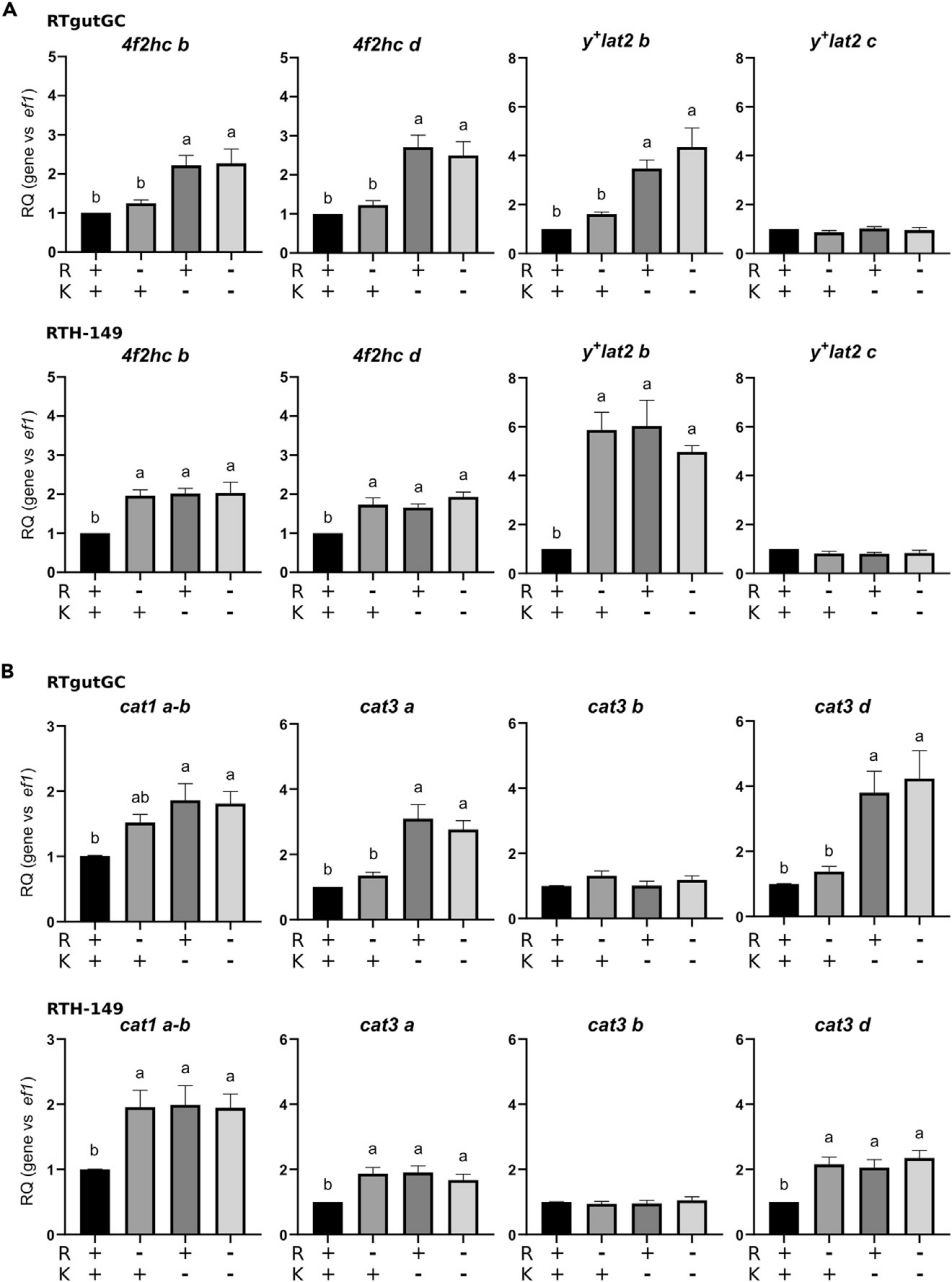
Expression of y^+^LAT and CAT genes upon arginine and/or lysine starvation Expression of y^+^LAT (A) and CAT (B) genes in arginine (R) and lysine (K) starvation in RTH-149 and RTgutGC cells. Cells were grown in arginine (R) and lysine (K) deprived medium supplemented (+) or not (−) with R and K prior to proceed to RNA extraction and RT-qPCR analysis of mRNA levels of ISR target genes. Results are represented as relative quotient (RQ) normalized on *ef1α* mRNA levels compared to condition with R and K. Data are presented as mean ± SEM, *N* = 6. Conditions showing results statistically different from each other are indicated using a different letter (p < 0.05, one-way ANOVA Tukey’s post-hoc test).

Since fish fed PBD supplemented with CAA displayed GCN2 activation (evidenced by the upregulation of *ddit3*) as well as the induction of CAAT paralogs (genes demonstrated from now on to be up-regulated upon AA starvation and/or GCN2 activation), we next questioned the effectiveness of the CAA supplementations mentioned to cover fish metabolic needs. To address this point, we therefore treated cells in media free of R and K (alone or in combination), containing dialyzed FBS, prior analyzing the outcomes of such starvations on the activation of the GCN2 pathway (Figure 4). Results showed that, when compared to RTH-149 cells, RTgutGC cells appeared to be completely insensitive to R starvation since none of the GCN2 target genes were up-regulated. On the other hand, K starvation led in both cell lines to the activation of the GCN2 pathway. Altogether, these results corroborated initial thoughts for which R and/or K starvation are enough stimuli to activate the GCN2 pathway together with highlighting different cell responses to R starvation between RTH-149 and RTgutGC cell lines. Of note, such difference in R cellular dependence remain to be elucidated since they cannot be explained by differences in gene expression from *de novo* arginine synthesis pathways (data not shown). Further investigations were therefore conducted to evaluate how RT CAAT paralogs were also subjected to transcriptional regulations in RT cell lines that showed different GCN2 activation patterns (qualitatively and quantitatively) to single or double CAA starvation conditions. Consistently with the previous results obtained, we observed in RTH-149 cells deprived from R that *4f2hc b*, *4f2hc d*, *y*^*+*^*lat2 b*, *cat1 a-b*, *cat3 a* and *cat3 d* expression levels were all significantly increased when compared to growing conditions containing R and K (Figure 5). Similarly, in RTgutGC cells which showed no sensitivity of the GCN2 pathway upon R starvation, none of these transporters were up-regulated. However, all these genes were shown to be up-regulated upon K starvation conditions in both cell lines (Figure 5B). Thus, in RTH-149 and RTgutGC cells, the CAAT expression patterns almost perfectly mirrored the GCN2 activation status measured upon single and double CAA starvation conditions. Finally, among the other CAAT paralogs assessed (Figure S5), only *ornt1 c* paralog, described to be regulated by starvation and HF treatments, showed consistent upregulations with respect for the GCN2 activation status measured in both cell lines. Altogether, these results clearly established correlations between RT CAAT paralogs, CAA restriction conditions and GCN2 activation which further demonstrates that part of the RT CAAT sub-family of genes is regulated by CAA availability in a GCN2 dependent manner. Moreover, *y*^*+*^*lat2 b* being the most up-regulated CAAT upon CAA starvation among the 16 CAAT assessed (Figure 5A), this result supported the hypothesis according which its overexpression in fish fed PBD, along with *ddit3* upregulation, might certainly reflect some AA-dependent nutritional limitations.

### CAA starvation impaired mTOR pathway activation but only K starvation led to defects in cell proliferation

**Figure 6:**
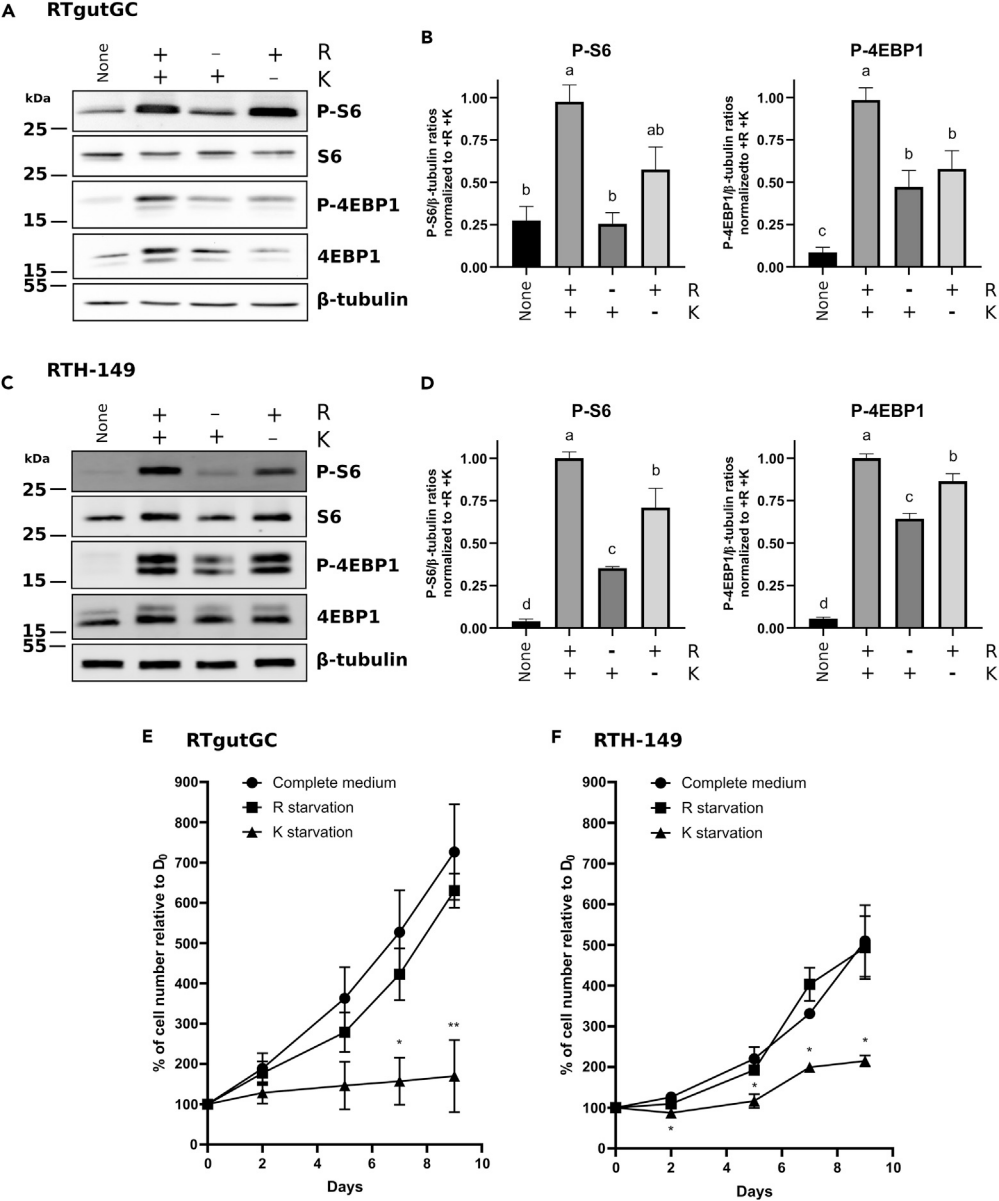
Arginine and lysine dependent regulations of mTOR pathway activation and cell proliferation (A–D) RTH-149 and RTgutGC cells were first starved in HBSS media before being treated for 5 h with medium deprived of serum, arginine (R) and lysine (K) supplemented (+) or not (−) with R and K prior protein extraction. Western blots were performed using antibodies directed against phospho-S6, phospho-4EBP1, total S6 and 4EBP1 proteins and β-tubulin as loading control. Quantifications of phospho-S6 and phospho-4EBP1 in RTgutGC (B) and RTH-149 (D) cells were performed using ImageJ software. Ratio between phosphorylated proteins and β-tubulin were calculated before normalization to +R +K condition. Data are presented as mean ± SEM, *N* = 3 (D) and *N* = 6 (A). Conditions showing results statistically different from each other are indicated using a different letter (p < 0.05, one-way ANOVA Tukey’s post-hoc test). (E and F) Cells were grown in culture medium deprived of arginine (R) and lysine (K) supplemented with with R (K starvation), K (R starvation) or both (Complete medium). Cells were counted using Nexcelom NK2 Cellometer. Results are presented as percentage of cell number counted for each condition normalized on cell number counted before treatment (D_0_). Results are represented as mean ± SEM, *N* = 3. Conditions showing, at each time point, results statistically different from complete medium condition are indicated as ∗: p < 0.05; ∗∗: p < 0.01; one-way ANOVA Dunnett’s post-hoc test).

Since all the results obtained so far corroborated our working hypothesis, outcomes of CAA restriction on RT cell lines were assessed at molecular and cellular levels. To do so, the mTOR pathway, a key anabolic signaling pathway controlling organism size and weight received a particular attention. Indeed, the activation of this serine/threonine kinase, known to promote lipid, carbohydrate, nucleotide and protein synthesis to sustain cell growth and proliferation, has been shown to be regulated by AA availability as well as AA transporters.^33^^,^^34^^,^^35^ Considering the differences observed between cell lines for their GCN2 responses following single R or K starvation, mTOR activation levels were assessed upon similar starvation conditions and compared to the levels measured in presence of both CAA (Figure 6). Western blot analysis revealed that, when cells were grown in media containing all AA, including R and K (+R + K), phosphorylation levels of S6 and 4EBP1 proteins, two mTOR targets, were significantly increased compared to levels measured upon starvation condition (None) (Figures 6A–6D). Interestingly, RTH-149 and RTgutGC cells only deprived of R showed a marked decrease in Phospho-S6 level and moderate, but still significant, decrease of Phospho-4EBP1 levels indicating that R promotes mTOR activation in these RT cell lines (Figures 6A–6D). It is important to notice that if phospho levels of these two mTOR activation markers do not drop to levels observed upon starvation conditions, it could be explained by the presence of other AA known to regulate mTOR activation into the media as well as by differences of the AA-induced phosphorylation and dephosphorylation kinetics of S6 and 4EBP1 already described in RTH-149 cells.^26^ Moreover, despite that unique K starvation in these two cell lines induced a moderate/intermediary effect on mTOR activation when compared to R starvation, it nonetheless suggested that, in RT species, K could also be sensed by the mTOR regulators. Altogether, these results showed, for the first time in trout, that both CAA are sensed by the mTOR pathway and positively contribute to its activation in different proportions depending on the CAA and cell lines considered.

Finally, based on all the data accumulated so far, we wondered whether such CAA deprivation would profoundly influence the physiology of the cells, in particular with regard to their capacity to proliferate, a characteristic that could explain the delay in the growth of fish fed with PBD. Accordingly, cell proliferation curves were established for 9 days while cells were grown in R and K free media supplemented or not with R and/or K. As a result, RTgutGC (Figure 6E) and RTH-149 (Figure 6F) cells did not shown any proliferative delay when deprived from R, compared to cell proliferation measured upon full AA containing media. On the other hand, K starvation strongly and significantly impaired cell proliferation which was limited to slight increase, certainly caused by the minimal supply of AA contained in fetal bovine serum. These observations, later discussed, were particularly stunning since both CAA were described as EAA in RT while R appeared as an NEAA for both cell lines in the tested conditions. At this stage, and since cell proliferation assays failed to be conducted using dialyzed serum to prevent the supply of cells with AA contained in serum, the ability of the two cell lines to produce R from the R *de novo* synthesis pathway, while being genetically equipped and expressing the enzymes from this pathway (data not shown), remains to be addressed.

### An *in vivo* pilot experiment as a proof of concept to improve supplemented CAA absorption in fish

**Figure 7:**
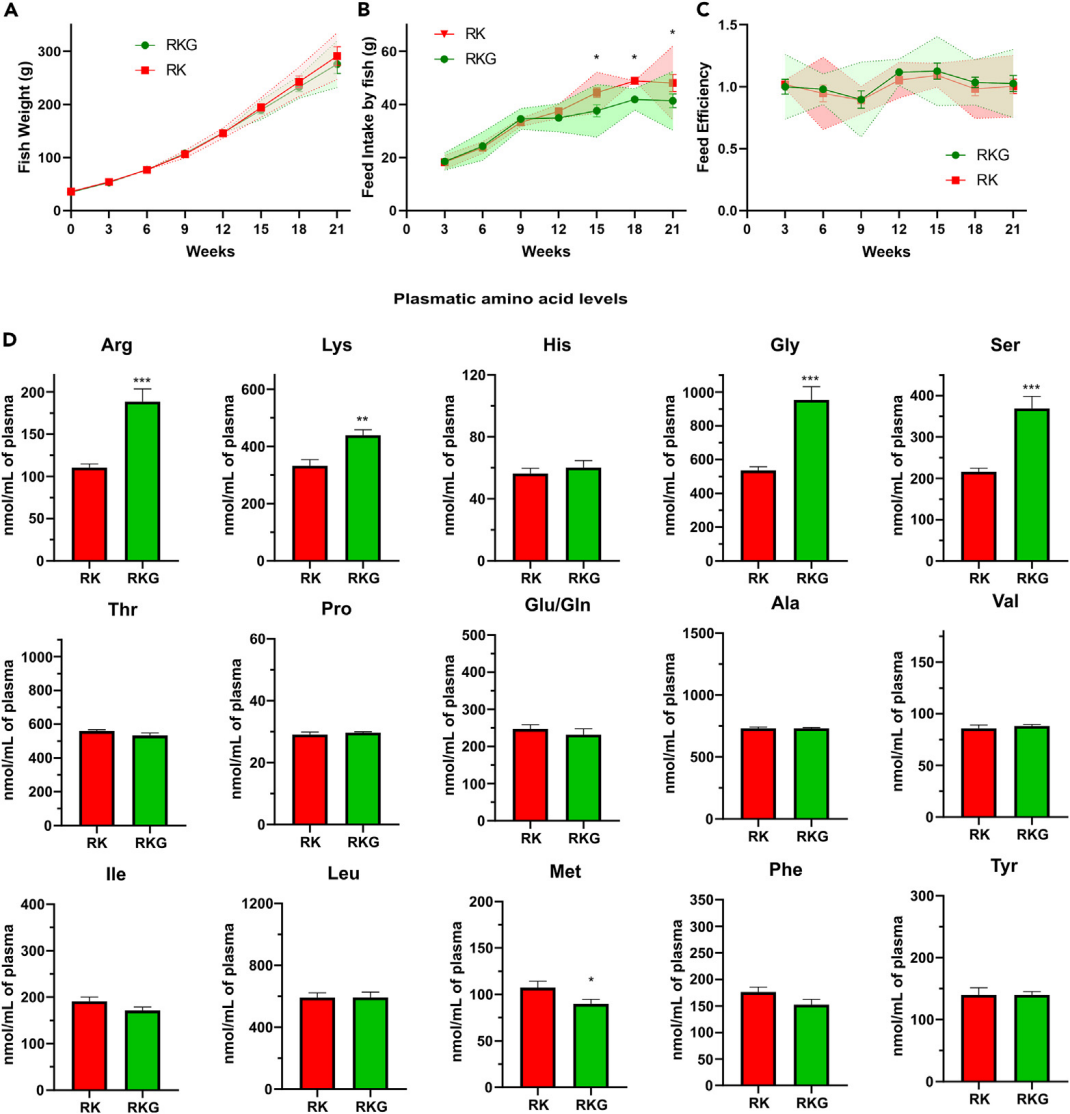
Effect of free glycine supplementation in plant-based diet already containing free arginine and lysine on fish growth performances and post-prandial plasmatic amino acid levels Fish were fed during 21 weeks with full plant-based diet enriched with free L-arginine and L-lysine supplemented with free glycine (RKG) or not (RK). Fish weights (A), feed intakes (B) and feed efficiencies (C) per fish were calculated for each diet every 3-week periods with gathered results obtained in the 3 tanks. Data are presented as mean of the values obtained at each period of 21 days ±SEM with 95% confidence intervals in their respective colors. Significant differences between diets are indicated as ∗: p < 0.01, two-way ANOVA with Sidak’s multiple comparisons test. Plasmatic amino acid concentrations sampled from caudal vein 5 h following fish feeding on the last day of growth trial (D) were analyzed by UPLC. Data are presented as mean ± SEM, *N* = 9. Significant differences between diets are indicated as ∗: p < 0.05; ∗∗: p < 0.01; ∗∗∗: p < 0.001; unpaired two-tailed Student test.

Since all the results obtained so far demonstrated that CAAT can be overexpressed in response to AA and CAA starvation, in a very likely GCN2 dependent manner, we pursued to challenge our hypothesis according which the overexpression of *y*^*+*^*lat2*, *cat2* and *ddit3* genes in fish fed PBD could be due to an absorption defect of the free CAA supplemented in these diets. Thus and based on the facts that (1) *y*^*+*^*lat2 b* paralog was the most dysregulated CAAT upon CAA starvation and (2) *y*^*+*^*lat* sub-family members, described for being strict exchangers, are, to the best of our knowledge, the only CAATs known to promote the CAA flux from the intestinal barrier to the blood, we tried a new formulation strategy to promote their absorption. Accordingly, we wondered whether supplementing PBD with a small and neutral amino acid, known in mammals to allow the exchange of CAA, would promote the activity of *y*^*+*^*lat* transporters to stimulate the absorption of supplemented R and K, and thus their bioavailability in the plasma of fish. To address this question, a particular attention was brought to glycine (G). Although G has not been extensively studied in CAAT-related mechanistic transport studies, it is noteworthy mention that our preliminary *in vitro* results tend to reveal that G would be one of the best neutral NEAA to promote R exchange (data not shown). Moreover, G supplementation of diets containing R and K in their free forms has been described to improve feed efficiencies in the species studied.^36^^,^^37^ Therefore, two diets were formulated and prepared with the exact same quantities of plant ingredients, requiring therefore R and K supplementations, while only one diet was also supplemented with 3% of G. Thus, diets were named according to their content in free AA as following: RK and RKG diets (Table 2). Then, diets were analyzed notably for their amino acid profiles (Table S2) to confirm that only G profiles differed from both diets prior to feed fish for 21 weeks. During this feeding trial, fish were bulk weighted every 3 weeks as well as the quantity of each feed distributed per tank to calculate feed intakes and feed efficiencies of both diets along the trial. Interestingly, while fish growth measured for both groups showed no difference over the 21 weeks’ trial (Figure 7A), a significant reduction of feed intake was observed during the second half of the trial with fish fed RKG diet compared to the RK group (Figure 7B) while this decrease in feed consumption did not affect feed efficiencies (Figure 7C). Since it is known that palatability issues of diets are usually detected by fish at first feedings, these first results tend to demonstrate that a G supplementation to the RK diet certainly induced a metabolic adaptation toward a better use of the diet which finally leads to 8% of diet saved in total for equivalent fish growth performance. In order to evaluate how much this improvement could be correlated to a better absorption of CAA, post-prandial AA profiles were determined during the aminoacidemia peak.^38^ Strikingly, and besides the obvious increase of plasmatic G, only serine (S), R and K concentrations displayed a marked and significant increase (Figure 7D). While plasmatic S increase could easily be linked to the increase of G (both amino acids are known to be metabolically connected through the activities of serine hydroxyl methyl transferase enzymes^39^), no clue was brought until now that the availability of plasmatic G promotes an increase in circulating CAA. According to our initial thoughts, it therefore seems that CAA absorption can be stimulated in fish fed PBD, constituting a first evidence that G would promote CAA fluxes to the blood. Moreover, when considering constant plasmatic histidine (H) levels measured from fish fed both diets, and knowing that it is an amino acid also uptaken by CAAT, it seems very likely to conclude that G supplementation specifically allowed the absorption of supplemented CAA, even if the precise mechanism by which G exerts this function remains unknown to date. Finally, these conclusions were also supported by the slight decrease in plasmatic methionine (M) levels observed in fish fed RKG diets. Indeed, despite being formally proved yet, there is growing body of evidence suggesting that part of M pool is taken over by CAAT. Accordingly, the increase of R and K fluxes might have slightly impaired M absorption, likely through a direct competitive inhibition that has yet to be confirmed. Altogether, results demonstrated that it is possible, in fish fed PBD, to specifically and precisely drive the absorption of supplemented CAA, certainly through the stimulation of *y*^*+*^*lat* transporters activities. Thus, such minimal modification of the diet formulation would therefore offer new feeding strategies of great importance for the aquaculture field, but not only, as later discussed.

## Discussion

This study was initiated following the observations that PBD induce dysregulations in RT, notably of CAAT genes, while these dysregulations could be related to unbalanced plant protein AA compositions although RT nutrient requirements are fulfilled by supplementing free CAA to PBD. Therefore, the full set of CAAT genes was identified for the first time in the RT complex genome, allowing subsequently, the comprehensive identification of CAAT genes expressed in trout tissues. Consequently, the nutritional regulations of CAAT paralogs according to AA- and CAA-specific availability were determined by means of convenient *in vitro* approaches using two independent RT cell lines. Results revealed that a sub-set of CAAT genes is very likely subjected to GCN2 pathway regulations whether pharmacologically activated or following AA specific- or CAA-specific-starvation conditions. The study of single CAA starvation conditions demonstrated that cells do not respond in similar ways to R or K starvation in terms of GCN2 activation and mTOR inhibition. Indeed, when K starvation induced GCN2 activation and moderated mTOR inhibition in both cell lines tested, almost opposite conclusions could be drawn from R starvation condition which led to a strong inhibition of mTOR pathway while the GCN2 pathway was not activated in RTgutGC cell line tested. Moreover, outcomes of R or K single starvation on cellular proliferation revealed that cells were not affected by R starvation while K starvation has a strong inhibitory effect on cell proliferation. Finally, this study demonstrates that improvements can still be performed in PBD formulation containing free R and/or K notably through an additional G supplementation to promote CAA absorption. Such G supplementation seems to favor dietary metabolization by RT as exemplified by the observed feed efficiency increase in RKG group compared to the regular RK diet. As a whole, this study further deepens our understanding of dysregulations induced by alternative diets used to feed farmed fish. Indeed, while most studies considered that supplementing PBD with purified AA is sufficient to cover AA theoretical needs of fish, the combination of data gathered from this study tends to demonstrate that PBD still need further improvements to definitively prevent AA-related dysregulations.

For instance, according to results obtained within single CAA starvation conditions, future experiments determining the best R:K ratio for fish requirement, instead of considering CAA individually, will be of great interest. Indeed, results obtained tend to demonstrate that R controls more the activation of anabolic process (notably through mTOR activation) than K does, while the lack of K would be more prone to activate catabolic and survival processes (notably through GCN2 pathway). As a consequence, knowing that R and K compete for their uptake with the same CAAT, not considering R:K ratios in PBD could result in the loss of homeostasis that is normally preserved following a tight balance between anabolism and catabolism. This statement is further supported by other negative side effects observed in fish fed plant meals such as hypercholesterolemia,^40^ described in other species to be regulated by CAA ratios^41^ and CAAT,^42^ as well as diabetes-like phenotype also governs in species,^43^ including trout.^44^, by CAA and AAT. Altogether, the use of RT cell lines appears as a credible model to study the outcomes of unbalanced AA composition of low-quality protein, such as plant proteins, on nutritional-induced dysregulations in fish but also in humans since both species display similar disorders when AA requirements are not fully covered.

Also, results gathered from this study prompt us to open a new field of investigation which could be termed “precision formulation” and which could be a good complement to “personalized nutrition” also called “precision nutrition”.^45^ Thus, “precision formulation” could be defined by the elaboration of a diet balanced for the different macro- and micronutrients, not only by supplementing ones missing, but also by providing molecules such as some AA, which, on the basis of fundamental knowledge gathered from mechanistic studies, optimize at best nutrient assimilation especially for those supplemented. Therefore, in a context of global limitation of protein supply for both human and animal nutrition, supposed to become critical in a very close future, “precision formulation” could improve feed efficiency and to reduce the amount of food required to properly feed the global population. Moreover, such strategy could seek to offer a larger panel of ingredients, even those poorly considered currently due to highly imbalanced AA profiles.

Finally, the use of RT cell lines to decipher molecular events related to fish nutrition, besides to prove being a powerful tool to identify and study nutritional regulation of trout gene expression, also brought insightful observations that could be of great interest for other fields such as medical sciences. Indeed, “precision nutrition” strategy that emerged from this study might also allow to adapt human diets to overcome some specific genetic disorders. For instance, lysinuric protein intolerance (LPI), an inherited rare disease caused by mutations in SLC7A7 gene (coding for the CAAT called y^+^LAT1) is notably known to considerably decrease the CAA flux across the intestine. The idea of using “precision formulation” strategy in such disease would be to specifically re-route CAA fluxes, via optimized dietary AA supplementation, through the activity of y^+^LAT2 (the other CAAT in charge of CAA fluxes at the basolateral membrane of enterocytes). Indeed, it is known that in such disease, y+LAT2, which is not mutated in patients, does not compensate the loss of y^+^LAT1. It seems very likely that such compensation does not operate in this pathology because of differences of y+LAT2 activities and specificities for AA exchanged compared to y^+^LAT1. Thus, supplementing diets of patients with some NEAA, such as G, to stimulate CAA fluxes from y+LAT2 activity could represent a new nutritional strategy for patients suffering from LPI-related physiological disorders.

Altogether, these results not only provide useful knowledge about the biology of trout as well as strategy to improve current PBD formulations required to sustain carnivorous fish production from the aquaculture field, but, more broadly, they also offer new models and open new avenue related to various pathologies such as hypercholesterolemia, diabetes, and inherited rare diseases caused by AAT mutations.

### Limitations of the study

A range of arguments, raised from our study, demonstrate that the bioavailability of CAA supplemented in their free forms to PBD can be improved by an extra supplementation of the PBD diet using G. Nonetheless, it is impossible at this stage to formally demonstrate that the molecular mechanisms driving this improvement rely on CAA exchanges mediated by y+LAT transporters in rainbow trout. As indicated in the article, we began to study the intrinsic AA transport properties and activities of the trout cells used in this article. The first preliminary results, which still need to be validated by experiments to silence the expression of the various y+LAT transporters, indicate that glycine is an amino acid that favors arginine exchange. However, these results cannot demonstrate that the effect observed *in vivo* depends mainly on the transport mechanisms of the y+LAT exchangers (in particular because the cell lines used do not express all the transporters found in trout tissues). To confirm this hypothesis, it would be interesting to establish at least 4 knock-out fish lines for each y+LAT1 and y+LAT2 paralogs (described in our study to be expressed in trout tissues) prior back-crossing these lines to establish the different combinations of y+LAT KO fish. If such genetic invalidations did not induce synthetic lethality in RT, it would be helpful to determine if G could promote the CAA fluxes through y+LAT transporters in RT.

## STAR★Methods

### Key resources table

**Table:** 

REAGENT or RESOURCE	SOURCE	IDENTIFIER
**Antibodies**		

Anti-ribosomal protein S6	Cell Signaling Technologies, Danvers, MA, USA	Cat#2217
anti-phospho-S6 (Ser235/Ser236)	Cell Signaling Technologies, Danvers, MA, USA	Cat#4856
Anti-phospho-4EBP1	Cell Signaling Technologies, Danvers, MA, USA	Cat#9459
Anti-4EPB1	Cell Signaling Technologies, Danvers, MA, USA	Cat#9452
Anti-β-tubulin	Cell Signaling Technologies, Danvers, MA, USA	Cat#2146
HRP-labeled goat anti-rabbit IgG secondary antibody	Thermo Fisher scientific, Waltham, MA, USA	Cat#31460
IRDye goat anti-rabbit secondary antibody	LI-COR, Inc., Lincoln, NE, USA	Cat#926-68071; RRID: AB_10956166

**Chemicals, peptides, and recombinant proteins**		

Minimum Essential Medium (MEM)	Thermo Fisher scientific, Waltham, MA, USA	Cat#61100-053
Phosphate Buffer Saline	Fisher Bioreagents, Fisher scientific SAS, Illkirch Graffenstaden, France	Cat#2944-100
Hank’s Balanced Salt Solution (HBSS)	Thermo Fisher scientific, Waltham, MA, USA	Cat#14065-056
Leibovitz’s L-15 Medium	Thermo Fisher scientific, Waltham, MA, USA	Cat#11415-049
MEM amino acid solution	Thermo Fisher scientific, Waltham, MA, USA	Cat#11130-036
MEM nonessential amino acid solution	Thermo Fisher scientific, Waltham, MA, USA	Cat#11140-50
Fetal bovine serum	Thermo Fisher scientific, Waltham, MA, USA	Cat#10270-106
Dialyzed Fetal bovine serum	Thermo Fisher scientific, Waltham, MA, USA	Cat#(#26400-044
Sodium pyruvate	Thermo Fisher scientific, Waltham, MA, USA	Cat#11360-070
Penicilin/Streptomycin	Thermo Fisher scientific, Waltham, MA, USA	Cat#14065-056
HEPES	Fisher Bioreagents, Fisher scientific SAS, Illkirch Graffenstaden, France	Cat#BP299-1
R and K deprived MEM	Genaxxon Biosciences, Ulm, Germany	Cat#C4230.0500
R and K deprived L-15	Genaxxon Biosciences, Ulm, Germany	Cat#C4061.0500
D-glucose	Sigma-Aldrich, Darmstadt, Germany	Cat#G8270
L-lysine	Sigma-Aldrich, Darmstadt, Germany	Cat#L5501
L-arginine	Sigma-Aldrich, Darmstadt, Germany	Cat#A5006
L-glutamine	Thermo Fisher scientific, Waltham, MA, USA	Cat#250030-081
Glycine	Sigma-Aldrich, Darmstadt, Germany	Cat#G7126
Halofuginone hydrobromide	Sigma-Aldrich, Darmstadt, Germany	Cat#32481
Tunicamycin	Sigma-Aldrich, Darmstadt, Germany	Cat#T7765
RIPA Buffer	Thermo Fisher scientific, Waltham, MA, USA	Cat#89901
Halt protease and phosphatase inhibitor cocktail	Thermo Fisher scientific, Waltham, MA, USA	Cat#78442
Water, Molecular Biology Grade	Fisher Bioreagents, Fisher scientific SAS, Illkirch Graffenstaden, France	Cat#BP2819-1

**Critical commercial assays**		

RNeasy Mini Kit	Qiagen, Hilden, Germany	Cat#74104
Superscript III RNAseH-reverse transcriptase kit	Invitrogen, Carlsbad, CA, USA	Cat#18080-093
Light Cycler 480 SYBR® Green 1 Master	Roche, Bale, Switzerland	Cat#04887352001
Bicinchoninic Acid Kit	Sigma-Aldrich, Darmstadt, Germany	Cat#BCA1-1KT
ViaStain^TM^ AOPI Staining Solution	Nexcelom Bioscience LLC Lawrence, MA, USA	Cat#CS2-0106
SupersignalTM West Pico Plus Chemiluminescent Substrate	Thermo Fisher scientific, Waltham, MA, USA	Cat#34580
AccQTag kit	Waters®, Saint-Quentin-en-Yvelines, France	Cat#186003836
AccQTagUltra A	Waters®, Saint-Quentin-en-Yvelines, France	Cat#186003838
AccQTagUltra B	Waters®, Saint-Quentin-en-Yvelines, France	Cat#186003839

**Experimental models: Cell lines**		

Rainbow trout: RTH-149 cells	ATCC®, LGC standards, Molsheim, France	Cat#CRL-1710
Rainbow trout: RTgutGC cells	Informally shared^46^	EAWAG (K. Schirmer)

**Experimental models: Organisms/strains**		

Rainbow trout	INRAE, Donzacq, France	

**Oligonucleotides**		

Random primers	Promega, Madison, WI, USA	Cat#C118A
Specific primers for analyzed genes (Table S1)	Eurogentec, Seraing, Belgium	Not applicable

**Software and algorithms**		

Primer 3	Untergasser A, Cutcutache I, Koressaar T, Ye J, Faircloth BC, Remm M and Rozen SG. Primer3--new capabilities and interfaces.^46^ Nucleic Acids Res. 2012 Aug 1; 40(15):e115.	https://primer3.ut.ee/
ImageJ	Rasband, W.S., ImageJ, U. S. National Institutes of Health, Bethesda, Maryland, USA^47^	https://imagej.nih.gov/ij/
Metabolite analysis software	Waters®, Saint-Quentin-en-Yvelines, France	Empower™ Pro software 3.0

**Other**		

Polyvinylidene fluoride (PVDF) membrane	Merck Millipore, Burlington, MA, USA	Cat#IFPL00010
RestoreTM PLUS Western Blot Stripping Buffer	Thermo Fisher scientific, Waltham, MA, USA	Cat#4630
Cellometer K2	Nexcelom Bioscience LLC	Cat#VisionLB-004-1117
Amicon Ultra-0.5 Centrifugal Filter Unit column (3kDa)	Merck Millipore, Burlington, MA, USA	Cat#UFC5003
Acquity H-Class PLUS (CH-A)	Waters®, Saint-Quentin-en-Yvelines, France	Cat#176015101
Acquity UPLC Fluo Detector	Waters®, Saint-Quentin-en-Yvelines, France	Cat#176015101
AccQTagUltra column	Waters®, Saint-Quentin-en-Yvelines, France	Cat#186003837
Odyssey® Imaging System	LI-COR, Inc	Cat# ODY-1647
iBright 1500 imager	Thermo Fisher scientific, Waltham, MA, USA	Cat# FL1500
Nanodrop	Thermo Fisher scientific, Waltham, MA, USA	ND1000 UV/vis
Light cycler 480	Roche, Bale, Switzerland	Cat#05015243001
Verity 96well thermal cycler	Thermo Fisher scientific, Waltham, MA, USA	Cat#4375786

### Resource availability

#### Lead contact

Further information and requests for resources and reagents should be directed to and will be fulfilled by the Lead Contact: Florian Beaumatin (florian.beaumatin@inrae.fr).

#### Materials availability

This study did not generate new unique reagents.

#### Data and code availability

• All data reported in this paper will be shared by the lead contact upon request.
• This paper does not report original code.
• Any additional information required to reanalyze the data reported in this paper is available from the lead contact.

### Experimental model and subject details

*In vitro* experiments were performed in two cell lines derived from rainbow trout tissues. One, the RTH-149 cell line, from liver and the other, RTgutGC cell line, from enterocytes to understand the effects of arginine and lysine starvations on CAAT expression, GCN2 and mTOR pathways as well as cell proliferation on trout liver and intestine. RTH-149 was generated from rainbow trout hepatoma cells induced with aflatoxin. We cultured RTH-149 from ATCC (ATCC CRL-1710, LGC standards, Molsheim, France) in complete Minimum Essential Medium (MEM) made with MEM powder containing L-glutamine (#61100-053), MEM nonessential amino acid (NEAA) solution (#11140-50), 10% fetal bovine serum (FBS, #10270-106), 10-mM sodium pyruvate (#11360-070), 100-units/mL penicillin and 100-μg/mL streptomycin (#14065-056), all from Gibco (Thermo Fisher scientific, Waltham, MA, USA), and 25-mM HEPES (#BP299-1, Fisher Bioreagents, Fisher scientific SAS, Illkirch Graffenstaden, France).

The other cell line, RTgutGC cells, gently provided by the Eawag–Swiss Federal Institute of Aquatic Science and Technology in Dübendorf, Switzerland, was grown in Leibovitz’s L-15 Medium (#11415-049, Gibco). This L-15 medium was supplemented with 100-units/mL penicillin and 100-μg/mL streptomycin (Gibco, #140656-056), 25 mM HEPES (#BP299-1, Fisher Bioreagents, Fisher scientific SAS, Illkirch Graffenstaden, France) and 10% fetal bovine serum (Gibco, #10270-106).

Both media, MEM and L-15 media are referred hereafter as complete medium (CM) respectively for RTH-149 and RTgutGC cells. RTH-149 and RTgutGC cells were grown at 18°C and medium replaced twice a week. Cell passages were done when they reached 80–90% of confluence for amplification and preparation of experiments.

For *in vivo* growth trial performed, rainbow trout were reared in INRAE NuMeA fish facilities of Donzacq (permit number A64.495.1 delivered by French veterinary services) for 21 weeks in 3 tanks per diet at a density of 21 fish per tank of 60 L at the beginning of the experiment before their transfer to 150 L tanks according to the weight of animals. Fish were fed twice a day *ad libitum* and amount of distributed feed was calculated every 3 weeks as well as mean fish weight and feed efficiency for each tank. The experiment was conducted according to the guidelines of the national legislation on animal care of the French Ministry of research (Decree No 2013-118, 1 February 2013). All efforts were made to reduce the number of fish used during the experiment as well as their suffering.

### Method details

#### Cell culture and treatments

Experiments were performed in hepatic cell lines RTH-149 (ATCC CRL-1710, LGC standards, Molsheim, France) and in the intestinal cell line RTgutGC. RTH-149 cells were grown at 18°C and pH 7.4 in complete medium (CM) as previously described.^48^ RTgutGC cells were grown in Leibovitz’s L-15 Medium containing essential (EAA), non-essential amino acids (NEAA) and sodium pyruvate from (11415-049) from Gibco (Thermo Fisher scientific, Waltham, MA, USA). This L-15 medium was supplemented with 100-units/mL penicillin and 100-μg/mL streptomycin (Gibco, #140656-056), 25 mM HEPES (#BP299-1, Fisher Bioreagents, Fisher scientific SAS, Illkirch Graffenstaden, France) and 10% fetal bovine serum (Thermo Fisher scientific, #10270-106). This medium is hereafter referred as CM for RTgutGC cell growth.

Medium was replaced twice a week and cells were passaged at 80–90% of confluence. Biological replicates for each experiments were performed with different passages. Prior to experiments, cells counted with Cellometer K2 (Nexcellom Bioscience LLC, MA, USA) and plated in 6 cm diameter dishes for western blot (WB) and real time quantitative PCR (RT-qPCR) analysis and in 12 well plates from Sarstedt (Sarstedt, Marnay, France) for cell proliferation assays. RTH-149 and RTgutGC were plated at a density of 400,000 cells for WB and 500,000 cells for RTq-PCR. For cell proliferation assays, cells were plated at a density of 70,000 for RTH-149 and 40 000 for RTgutGC per wells in 3 technical replicates per condition and time point. Two days after seeding, cells were washed twice with PBS (#2944-100, Fisher bioreagents) prior treatment according to experiments. In RT-qPCR experiments assessing effect of amino acids on gene transcription, cells were treated with CM or Hank’s balanced salt solution (HBSS) (#14065-056, Thermo Fisher) supplemented with 25 mM HEPES, supplemented (+AA) or not (-AA) with MEM amino acid solution containing all EAA (#11130-036), NEAA solution (#11140-050) and L-glutamine (#250030-081, Thermo Fisher). Halofuginone hydrobromide (HF) (#32481, Sigma-Aldrich, Darmstadt, Germany), a pharmacological inhibitor of prolyl-tRNA synthetase was added to CM at indicated concentrations to activate the GCN2 pathway or not (CTRL). To assess the effects of arginine (R) and lysine (K) starvations on gene expression, we used media nominally free of R and K (#C4230.0500, Genaxxon Biosciences, Ulm, Germany) and L-15 (#C4061.0500, Genaxxon Biosciences). Both were supplemented with dialyzed FBS (#26400-044, Thermo Fisher scientific), sodium pyruvate, penicillin and streptomycin as for CM. C4230 medium was also supplemented with 1 g/L D-glucose (#G8270, Sigma-Aldrich) and 25 mM HEPES which are not included is this medium. 100 mg/L L-Lysine (#L5501, Sigma-Aldrich) was added to both media, L-arginine (#A5006, Sigma-Aldrich) was added or not at 84 mg/L in C4230 medium and 420 mg/L in C4061 to obtain our experimental conditions. For WB analysis, cells were starved for 3 h or 5 h (RTH-149 or RTgutGC cells respectively), with starvation medium to inactivate mTOR pathway prior treatments. Cells were treated with the same media described before for R and K starvation effect on gene expression but without addition of serum. For cell proliferation assays, C4061 medium supplemented with non-dialyzed serum (#10270-106, Thermo Fisher) was used for the 2 cell lines.

#### RNA extraction and RT-qPCR analyses

For gene expression analysis experiments, RTH-149 and RTgutGC were treated for 24h. Total RNA were extracted and purified, after two wash with PBS, using a RNeasy Mini Kit (Qiagen, Hilden, Germany) according to manufacturer’s protocol. RNA concentrations and integrities were assessed with a Nanodrop ND1000 spectrophotometer (Thermo Fisher scientific). 1 μg of RNA was retrotranscribed using Superscript III RNAseH-reverse transcriptase kit (#18080-093, Invitrogen, Carlsbad, CA, USA) with random primers (#C118A, Promega). Using a thermocycler (Verity 96well thermal cycler, #4375786, Thermo Fischer scientific) a first step of denaturation was performed (5 min at 65°C) followed by RNA retro-transcription (5 min at 25°C, 1 h at 55°C) and inactivation at 70°C during 15 min. RT-qPCR reactions were performed on the Light Cycler 480 system (#05015243001, Roche) initiated at 95°C for 10 min followed by 45 cycles of an amplification program constituted of three phases (15 s at 95°C, 10 s at 60°C, 15 s at 72°C). RT-qPCR reactions were performed in triplicates comprising 2 μL of diluted cDNA at 1/40 for CAAT genes and 1/80 for others, 3 μL of Light Cycler 480 SYBR Green 1 Master (#04887352001, Roche), 0.76 μL of Water, Molecular Biology Grade (#BP2819-1, Fisher Bioreagents) and 0.12 μL (10 μM) of specific primers to studied genes. Primers were designed using Primer3 software for each RT paralog when possible. Validation of primers was performed by assessing primer efficiencies by RT-qPCR in a pool of mRNA samples from liver, gut, muscle, kidney and ovary as well as a pool of mRNA from brain and hypophysis for SLC7A4 and SLC7A14 homolog genes. Primers were validated when i) efficiencies was comprised between 1,85 and 2,15, ii) RT-PCR product migrate in an agarose gel at the expected size and iii) when sequence obtained by DNA sequencing (Genewiz, Leipzig, Germany) corresponded to the theorical sequence of the mRNA targeted. Primers for *ddit3*, *asns*, *xbp1*, *edem1* and *slc7a7* validated in previous studies^26^^,^^28^^,^^49^^,^^50^^,^^51^ as well as those newly designed and validated for all RT CAAT genes identified are listed in Table S1. The gene expression levels were presented as the relative quotient (RQ) calculated using the ΔΔCT method.

#### Protein extraction and western blot analysis

Cells were washed twice with ice-cold PBS prior to protein extraction using RIPA buffer (#89901, Thermo Scientific) supplemented with Halt protease and phosphatase inhibitor cocktail (#78442, Thermo Scientific). Prior to centrifugation at 12,000x *g* at 4°C, proteins samples were conserved on ice during 30 min. Following protein extraction, determination of protein concentration in samples were performed using the Bicinchoninic Acid Kit (#BCA1-1KT, Sigma-Aldrich). Protein samples were mixed with Laemmli buffer and subjected to electrophoresis with sodium dodecyl sulfate polyacrylamide gel (SDS-PAGE) prior to transfer onto polyvinylidene fluoride (PVDF) membrane (#IFPL00010, Merck Millipore, Burlington, MA, USA). Membranes were finally immunoblotted using the following primary antibodies directed against β-tubulin (#2146) as a loading control and mTOR pathway targets: anti-ribosomal protein S6 (#2217), anti-phospho-S6 (Ser235/Ser236, #4856), anti-phospho-4EBP1 (Thr37/Thr46, #9459), anti-4EBP1 (#9452) all provided by Cell Signaling Technologies (Danvers, MA, USA). A part of the experiment analysis was performed using infrared fluorescence as described in our previous paper^48^ or using chemiluminescence. Briefly, IRDye secondary antibody (#926–68071, LI-COR, Inc., Lincoln, NE, USA) or HRP-labeled goat anti-rabbit IgG secondary antibody (#31460, Thermo Fisher Scientific) were incubated on membranes prior washes. Signal detection were ensured using the Odyssey Imaging System (LI-COR, Inc) or following membrane incubation in Supersignal West Pico Plus Chemiluminescent Substrate (#34580, Thermo Fisher Scientific) according to manufacturer’s protocol and signal acquired using iBright 1500 imager (Thermo Fisher Scientific). The other part was analyzed using chemi-luminescence. Membranes were incubated with and washed with PBS prior Immunoblot for total S6, 4EBP1 and β-tubulin were performed after membrane stripping with Restore PLUS Western Blot Stripping Buffer (#4630, Thermo Fisher Scientific). All WB data were quantified using ImageJ software (NIH, Bethesda, MD, USA). Data are presented as ratio of phosphorylated protein normalized on β-tubulin.

#### Cell proliferation assays

Two days after incubation of cells in CM (Day 0) cells were treated in the indicated growing condition and for different time points prior being washed with PBS, stained (ViaStain AOPI Staining Solution) and counted using Cellometer K2 (Nexcellom Bioscience LLC) following manufacturer’s indications. Counting was performed for 3 technical replicates with 3 individual wells for each condition and time point. For each biological replicate data are presented as the mean of cell numbers counted in technical triplicates normalized on the cell number counted at Day 0.

#### Feeding trial and post-prandial plasmatic amino acid concentrations

Fish around 20 g were reared for 21 weeks in 3 tanks per diet at a density of 21 fish per tank of 60 L at the beginning of the experiment before their transfer to 150 L tanks according to the weight of animals. Fish were fed twice a day *ad libitum* and amount of distributed feed was calculated every 3 weeks as well as mean fish weight and feed efficiency for each tank. Rainbow trout were fed two isoproteic and isoenergetic full plant-based diets covering dietary requirements of trout. One plant-based diet supplemented with free L-arginine and L-lysine at respectively 0,5 and 2,2 percent of the diet (RK) and the same diet also supplemented with 3% of free glycine (RKG) (#G7126, Sigma), the detailed compositions are available in Table 2. Analysis of amino acid profile of both diets by Upscience (Saint-Nolff, France) showed no differences between diets except for glycine levels which are more elevated in RKG diet as consequence of its supplementation in free form (Table S2). Plasma were sampled 5 h after the last meal of the trial. To this end, fish were euthanized before blood sampling from the caudal vein. Blood was collected with heparinized syringes and centrifugated 5 min at 3000x g. For plasma deproteinization, 200 μL of plasma were transferred to Amicon Ultra-0.5 Centrifugal Filter Unit column (3kDa) (#UFC5003, Merck Millipore) and then centrifugated 30 min at 12000 g at 4°C. The obtained filtrates were conserved at −20°C before analysis using a Acquity H-Class PLUS (CH-A) (Waters) Alliance System (2695 separation module) coupled to a Acquity UPLC Fluo Detector (Waters). Plasma filtrates were derivatized using AccQTag kit (#186003836, Waters, Saint-Quentin-en-Yvelines, France) following manufacturer’s instructions. 30 μL of borate buffer and 10 μL of AccQ Fluor derivatizing reagent were added to 10 μL of deproteinized plasma prior to vortexing and heating at 55°C for 10 min 10 μL of samples were separated during.^52^13 min using AccQTagUltra column heated at 49°C. The flow rate of the mobile phase was 0.7 mL/min. The mobile phase was a solvent system composed of 4 AccQTagUltra A (A), AccQTagUltra B/H2O ultrapure 1/9 (B), H2O ultrapure (C) and AccQTagUltra B (D) all filtered through 0.2-μm membrane filters. The separation gradient was initially set to 2.0% A and 98.0%, this condition is conserved for 0.29 min after sample injection, then 2.0% A and 98.0% C for 0.29 min, then 9.0% A, 80.0% B and 11.0% C for 5.49 min, 8.0% A, 15.6% B, 55.9% C and 20.5% D for 14.40 min, then 7.8% A, 70.9% C and 21.3% D for 7.69 min, then 4.0% A, 36.3% C and 59.7% D for 16.58 min and finally the initial conditions for 11 min. The fluorescence of the eluate was monitored at λexc set to 266nm and λem to 473 nm. Data acquisition was performed using Empower Pro software 3.0 (Waters). Amino acids were identified according to the retention time and m/z ratio compared to standards. Amino acid concentration was determined against a standard curve of 7 points (0–1000 μM).

### Quantification and statistical analysis

All values are presented as means ± S.E.M. Number of biological replicates (*N*) is indicated for each experiment in figure legends. *In vitro* experiments were performed at least 3 times with biological replicates performed with different cell passages.

The gene expression levels were presented as the relative quotient (RQ) to complete medium (CM) or control (CTRL), calculated using the ΔΔCT method with *ef1* as housekeeping gene. All WB data were quantified using ImageJ software (NIH, Bethesda, MD, USA). Data are presented as ratio of phosphorylated protein normalized on β-tubulin itself normalized on condition presenting the highest levels of phosphorylation. For cell proliferation, counting was performed for 3 technical replicates with 3 individual wells for each condition and time point. For each biological replicate data are presented as the mean of cell numbers counted in technical triplicates normalized on the cell number counted at Day 0.

Normality was assessed individually for each condition using Shapiro-Wilk test (p > 0.05). Then differences between conditions were tested using one-way ANOVA-Tuckey’s post-hoc test when datasets passed normality test or t-test when they were only 2 conditions in the experiment. For cell proliferation assays, one-way ANOVA Dunnett’s post-hoc test was used to see differences between R and K starvation conditions in comparison with complete medium. For multiple comparisons, letter code was used to ease the interpretation of the statistical analyses results. Thus, when statistical differences (p value <0.05) were observed between certain conditions, these conditions were affected different letters. On the other hand, conditions displaying similar letters should be considered statistically not different.

## Supplemental information

Document S1. Figures S1—S5

Table S1. List of real-time quantitative PCR (RT-qPCR) primers used in this study, related to figures 1, 2, 3, 4 and 5 Primers for Rainbow trout CAAT genes were newly designed for each paralog when possible or for different paralogs except for primers of *y*^*+*^*lat1 a* and *y*^*+*^*lat1 b* as well as for *ef1α*, ISR and UPR targets which were designed in previous studies. Primer efficiencies were tested by RT-qPCR with a pool of mRNA samples from selected tissues including liver, intestine, muscle, kidney and ovary and are indicated in the last column. Primer efficiencies for SLC7A4 and SLC7A14 paralogs were tested in different pool of mRNA samples extracted from brain. To validate primers, sizes of amplicons were verified through migration on agarose gel prior sequencing. After validation in RT tissues, expression of the different CAAT paralog genes were assessed separately in liver, whole gut as well as in RTH-149 and RTgutGC cell lines.

Table S2. Amino acid levels measured in the RK and RKG diets used in the feeding trial, related to figure 7 Theoretical AA needs of RT are indicated in the right column of the table (according to ^25^).
