# Supplementary material for: Precision formulation, a new concept to improve dietary amino acid absorption based on the study of cationic amino acid transporters
Source: iScience. 2024 Jan 14;27(2):108894. doi: 10.1016/j.isci.2024.108894 (PMC10839688; doi:10.1016/j.isci.2024.108894)
Supplement: Document S1. Figures S1—S5 [file mmc1.pdf]

iScience, Volume *27*

Supplemental information

Precision formulation, a new concept to improve dietary amino acid absorption based on the study of cationic amino acid transporters

Guillaume Morin, Karine Pinel, Cécile Heraud, Soizig Le-Garrec, Chloé Wayman, Karine Dias, Frédéric Terrier, Anthony Lanuque, Stéphanie Fontagné-Dicharry, Iban Seiliez, and Florian Beaumatin

Supplementary Figures

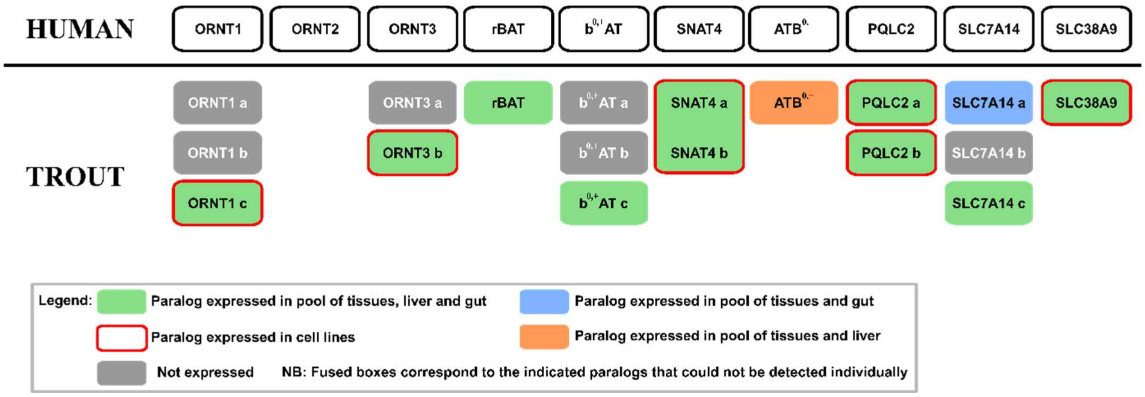

Figure S1 - **Human genes coding for the other CAAT and their ortholog genes identified in RT genome and expressed in RT tissues and in RTH-149 and RTgutGC cell lines.** 18 orthologs of the 10 other human CAAT-related genes were identified following *in silico* analysis of RT genome (Omyk 1.0). When possible, RT-qPCR primers were designed to discriminate each paralog. Primers that recognise more than one paralog are shown as fused boxes for the indicated paralogs. Gene expression was assessed in a pool of RT tissue samples (including liver, gut, muscle, kidney, ovary and brain) and in liver or gut samples separately as well as in the 2 RT cell lines. Gene expression analysis by RT-qPCR in RT cell lines revealed that they express the same CAATs which are also expressed in liver and gut

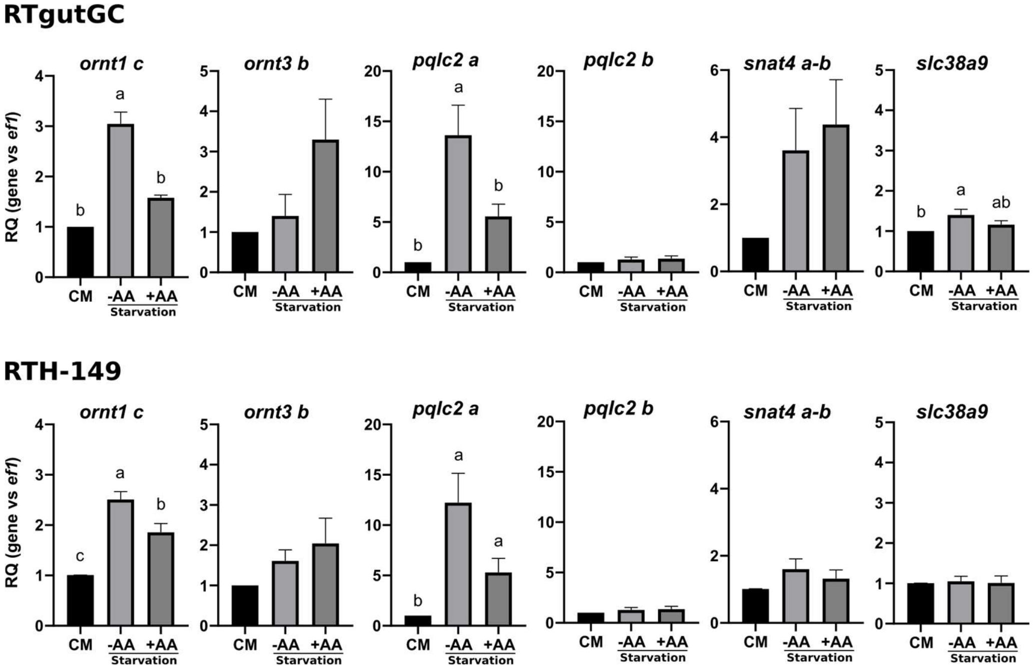

Figure S2. Amino acid dependent regulations of other CAATs in RTH-149 and RTgutGC cells. Cells were grown in either complete medium (CM) or in HBSS (starvation) supplemented (+AA) or not (-AA) with amino acids prior to proceed to RNA extraction and RT-qPCR analysis of mRNA levels of CAAT genes. Results are represented as relative quotient (RQ) normalized on *ef1α* mRNA levels compared to CM. Data are presented as mean ± SEM, $N = 6$ for RTH-149 cells and $N = 4$ for RTgutGC cells. Conditions showing results statistically different from each other are indicated using a different letter ($p < 0.05$, one-way ANOVA Tukey's post-hoc test).

CC BY

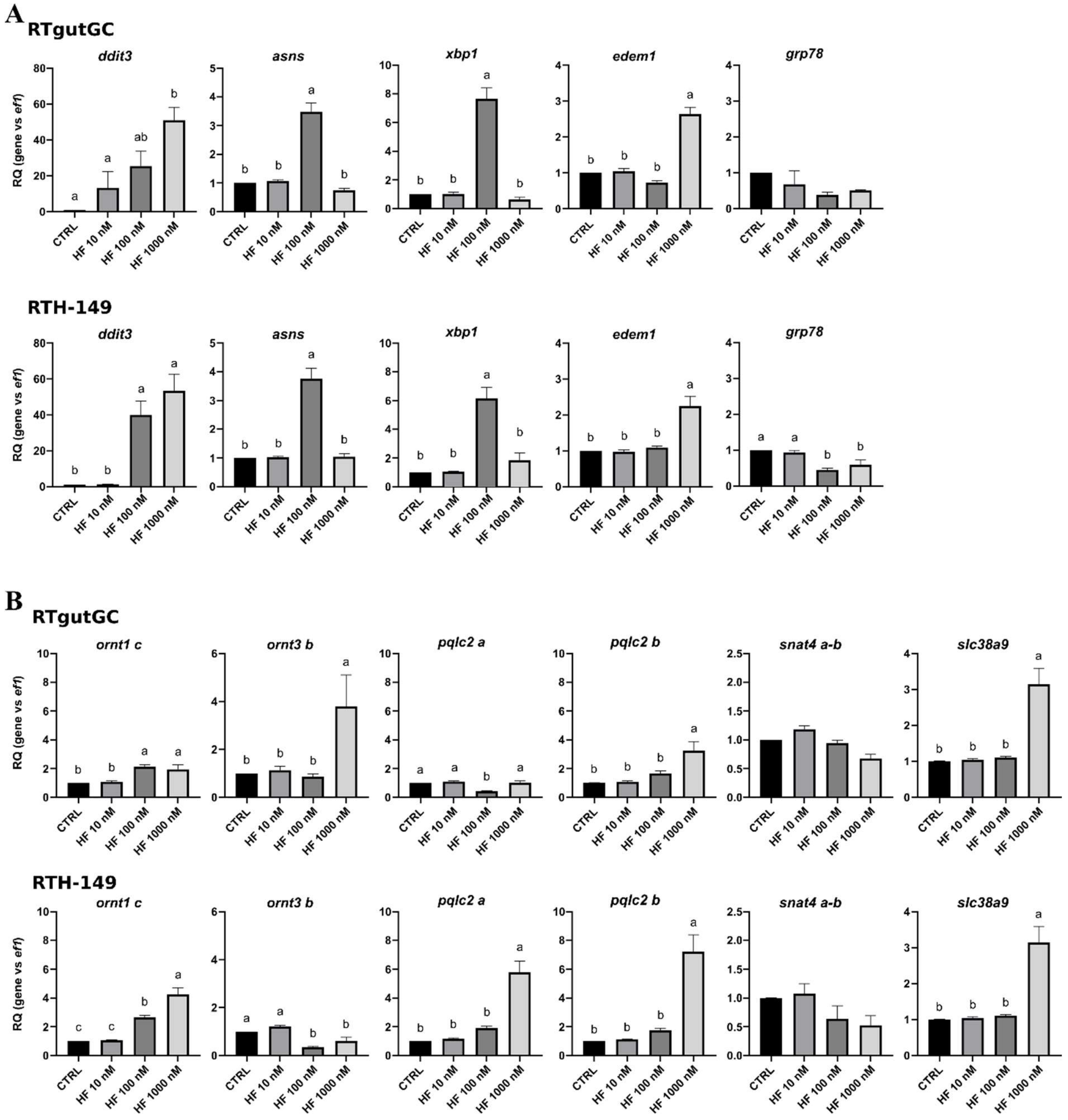

Figure S3. Halofuginone-induced ISR target genes and CAAT regulations in RTgutGC and RTH-149 cells. Cells were grown in complete medium supplemented with indicated concentrations of HF or without (CTRL) prior to proceed to RNA extraction and RT-qPCR analysis of mRNA levels of ISR target genes (A) or CAAT genes (B) in RTgutGC and RTH-149 cells. Results are represented as relative quotient (RQ) normalized on *ef1α* mRNA levels compared to CTRL. Data are presented as mean ± SEM, *N* = 4. Conditions showing results statistically different from each other are indicated using a different letter ($p < 0.05$, one-way ANOVA Tukey's post-hoc test).

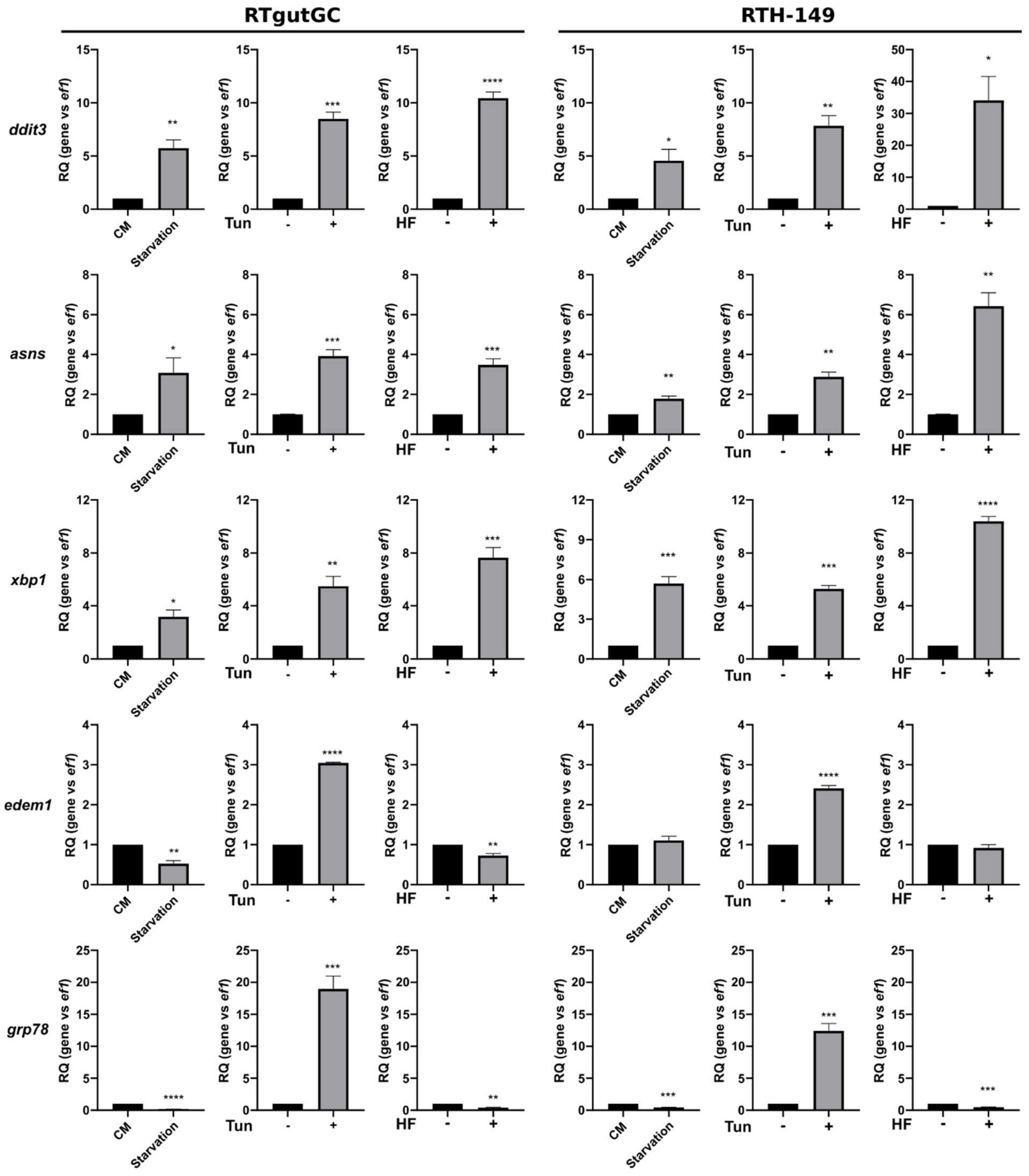

Figure S4. Expression of ISR and UPR target genes induced by starvation, tunicamycin or halofuginone hydrobromide in RTgutGC and RTH-149 cells. Cells were grown in starvation (HBSS) or complete medium (CM) supplemented (+) or not (-) with 1 µM tunicamycin (Tun) or 100 nM HF prior to extract RNAs and proceed to RT-qPCR analysis of ISR and UPR target genes. Results are represented as relative quotient (RQ) normalized on *ef1α* mRNA levels compared to complete medium. Data are presented as mean ± SEM, *N* = 3. Conditions showing results statistically different from each other are indicated are indicated as * : $p < 0.05$; ** : $p < 0.01$; *** : $p < 0.001$; **** : $p < 0.0001$; unpaired two-tailed student test.

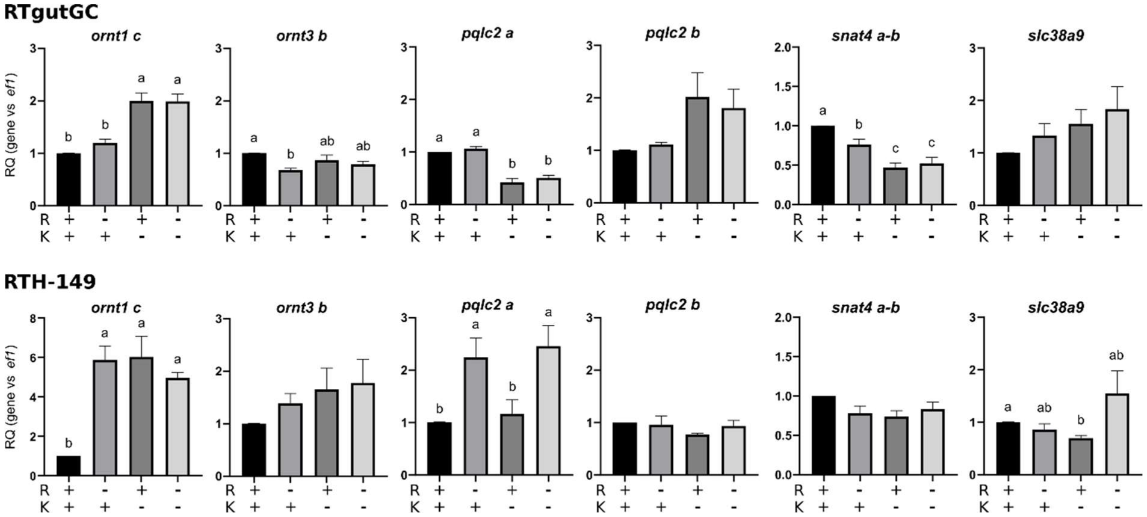

Figure S5. Arginine and lysine dependent regulations of the other CAATs in RTH-149 and RTgutGC cells. Cells were grown in arginine (R) and lysine (K) deprived medium supplemented (+) or not (-) with R and K prior to proceed to RNA extraction and RT-qPCR analysis of mRNA levels of ISR target and CAAT genes. Results are represented as relative quotient (RQ) normalized on *ef1α* mRNA levels compared to condition with R and K. Data are presented as mean ± SEM, *N* = 6 cells. Conditions showing results statistically different from each other are indicated using a different letter ($p < 0.05$, one-way ANOVA Tukey's post-hoc test).
