# Supplementary material for: Precision formulation, a new concept to improve dietary amino acid absorption based on the study of cationic amino acid transporters
Source: iScience. 2024 Jan 14;27(2):108894. doi: 10.1016/j.isci.2024.108894 (PMC10839688; doi:10.1016/j.isci.2024.108894)
Supplement: Table S1. List of real-time quantitative PCR (RT-qPCR) primers used in this study, related to figures 1, 2, 3, 4 and 5 — Primers for Rainbow trout CAAT genes were newly designed for each paralog when possible or for different paralogs except for primers of y+lat1 a and y+lat1 b as well as for ef1α, ISR and UPR targets which were designed in previous studies. Primer efficiencies were tested by RT-qPCR with a pool of mRNA samples from selected tissues including liver, intestine, muscle, kidney and ovary and are indicated in the last column. Primer efficiencies for SLC7A4 and SLC7A14 paralogs were tested in different pool of mRNA samples extracted from brain. To validate primers, sizes of amplicons were verified through migration on agarose gel prior sequencing. After validation in RT tissues, expression of the different CAAT paralog genes were assessed separately in liver, whole gut as well as in RTH-149 and RTgutGC cell lines. [file mmc2.pdf]

Genes	Forward Primer	Reverse Primer	Efficiencies
cat1 a and *cat1 b*	5’-GCTGCACTGTCAACATCACT-3’	5’-TGGTCTTGCTCTCTGGTTGT-3’	1,94
cat2 a and *cat2 b*	5’-GGTGCTCATCAACGCTACAG-3’	5’-ACAAATGAGGAGAGACGCCA-3’	2,11
cat2 c	5’-TCTCTGACGGTGTGCTTTCT-3’	5’-GTAAGCCATCTCGTGCCATG-3’	1,95
cat3 a	5’-GCGAACGTGAAGTCAACTGT-3’	5’-CAACAAGATCCGCCCAAAGG-3’	1,94
cat3 b	5’-CGGACTGTAAAGTTCAATCGGA-3’	5’-GGAGCAGCATCTTACCGAAG-3’	1,97
cat3 d	5’-ACACCAACACCTCCAGCATA-3’	5’-CCCGAAGTAGGCGAAGAAAC-3’	1,96
4f2hc b	5’-GGAGAGTGGTCGGAACAAGT-3’	5’-TCTCCTGGTCCAACTCGTTC-3’	2,00
4f2hc d	5’-CACCTCGCTGCAAAGACATT-3’	5’-TTGCCAACGTCAGAGGAGAT-3’	1,97
y^+lat1 a and *y^+lat1 b*	5’-GAGGACTCAACGCTTCTATC-3’	5’-CAACACACAGGTAGACCAA-3’	2,03
y^+lat2 b	5’-CCATCTCCATGCCCATTGTG-3’	5’-AGGCGTTGAGTCCTCCATAG-3’	2,0
y^+lat2 c	5’-GTATCGGGGTGGCTCTTTCT-3’	5’-TCCAGGTCTAGGTCAGGTGA-3’	1,97
rbat	5’-GAGGAGAGGCAGCAGTGTTA-3’	5’-AGGATCTTGTTGTGGGTCCA-3’	2,11
$b^{(0,+)}at1$ c	5’-GCCTACGATGGGTGGAATCA-3’	5’-CAAAAGTCACAGCAACAGCG-3’	1,88
atb^{0+}	5’-CCTATGTGGGCAGCAATGAT-3’	5’-TACGGGATGAGGAAAGCACC-3’	2,10
snat4 a and *snat4 b*	5’-TCCTTTGGTATGTCGGTCTTCA-3’	5’-GCAGTCATTAGCAGCAAGTGA-3’	2,09
slc38a9	5’-GTATTGACATAGTGCCTGCGG-3’	5’-GAGTCGGCTGTAGTAGTGCA-3’	1.98
ornt1 c	5’-ACGCTGTCCTTTTCATGAGC-3’	5’-GGACCACACCGAACTCTTCT-3’	2,01
ornt3 b	5’-ACATTGGACTTTGCAGCAGG-3’	5’-GGCGTTGATGAAGGTCAGAC-3’	2,07
pqlc2 a	5’-AGCAGTGTTTTGAGAGCGTG-3’	5’-TCCCAAACCCATACCGATCC-3’	2,01
pqlc2 b	5’-TTTGGTTTCTGCTGCTGTGG-3’	5’-GCAGAGAAGCCCAGGACATA-3’	2,10
cat4 a	5’-TCTCGTTCTCCATCTCCACC-3’	5’-TCACACTCGGCAACTTATCAG-3’	1,95
cat4 b	5’-CGCTTGTCTGATGGCTGATC-3’	5’-TCCATTCTTCTCCTGTTCGACT-3’	1,86
slc7a14 a	5’-TCTAATCTGCCAGGGTCACG-3’	5’-TATCCTTGTGAAGACGGGCT-3’	2,13
slc7a14 c	5’-CTGCCAGGGAAATACATAGTGG-3’	5’-ACATGAGAGCAGCCAGACAG-3’	2,05
ddit3	5’-CGACAATGTCCAACAACCTG-3’	5’-ACGAGGAGAACGAGGTGCTA-3’	1,97
asns	5’-CTGCACACGGTCTGGAGCTG-3’	5’-GGATCTCGTCTGGGATCAGGTT-3’	1,94
xbp1	5’-TGCAACCAAGCCAATTCTTC-3’	5’-GCGAGAACTTCGTCTTCCAG-3’	1,95
edem1	5’-GAACATCCAAACGGGACAGT-3’	5’-TGAGAAGAGGGAGGGAGTCA-3’	1,95
grp78	5’-CAACCCCGAGAACACAGT-3’	5’-GACCACAGCGTGTGTCACTT-3’	1,91
ef1α	5’-TCCTCTTGGTCGTTTCGCTG-3’	5’-ACCCGAGGGACATCCTGTG-3’	1,91
