# Supplementary material for: Precision formulation, a new concept to improve dietary amino acid absorption based on the study of cationic amino acid transporters
Source: iScience. 2024 Jan 14;27(2):108894. doi: 10.1016/j.isci.2024.108894 (PMC10839688; doi:10.1016/j.isci.2024.108894)
Supplement: Table S2. Amino acid levels measured in the RK and RKG diets used in the feeding trial, related to figure 7 — Theoretical AA needs of RT are indicated in the right column of the table (according to 25). [file mmc3.pdf]

**Amino acids g/100 g of dry diet**	**RK**	**RKG**	**Theoretical AA requirements (%)**
Aspartate	2.55 ± 0.20	2.37 ± 0.19	
Glutamate	10.07 ± 0.81	9.05 ± 0.72	
Alanine	3.48 ± 0.28	3.25 ± 0.26	
Arginine	1.91 ± 0.15	1.87 ± 0.15	1.5
Cystine	0.79 ± 0.06	0.76 ± 0.06	
Glycine	1.31 ± 0.10	4.10 ± 0.33	
Histidine	0.87 ± 0.07	0.82 ± 0.07	0.8
Isoleucine	1.63 ± 0.13	1.56 ± 0.12	1.0
Leucine	6.44 ± 0.52	5.60 ± 0.45	1.6
Lysine	2.55 ± 0.20	2.52 ±0.20	2.2
Methionine	1.02 ± 0.08	0.99 ± 0.08	0.7
Phenylalanine	2.51 ± 0.20	2.43 ± 0.19	0.9
Proline	4.27 ± 0.34	3.94 ± 0.32	
Serine	2.22 ± 0.18	2.15 ± 0.17	
Threonine	1.42 ± 0.11	1.37 ± 0.11	1.1
Tryptophan	0.28 ± 0.03	0.27 ± 0.03	0.3
Tyrosine	1.94 ± 0.16	1.89 ± 0.15	
Valine	1.88 ± 0.15	1.78 ± 0.14	
Total amino acids	46.88 ± 3.75	46.44 ± 3.72	
